# A Review on Measures to Rejuvenate Immune System: Natural Mode of Protection Against Coronavirus Infection

**DOI:** 10.3389/fimmu.2022.837290

**Published:** 2022-03-15

**Authors:** Md. Aminul Islam, Md. Atiqul Haque, Md. Arifur Rahman, Foysal Hossen, Mahin Reza, Abanti Barua, Abdullah Al Marzan, Tuhin Das, Sumit Kumar Baral, Cheng He, Firoz Ahmed, Prosun Bhattacharya, Md. Jakariya

**Affiliations:** ^1^Department of Microbiology, Noakhali Science and Technology University, Noakhali, Bangladesh; ^2^Department of Microbiology President Abdul Hamid Medical College, Karimganj, Bangladesh; ^3^Key Lab of Animal Epidemiology and Zoonoses of Ministry of Agriculture and Rural Affairs, College of Veterinary Medicine, China Agricultural University, Beijing, China; ^4^Department of Microbiology, Faculty of Veterinary and Animal Science, Hajee Mohammad Danesh Science and Technology University, Dinajpur, Bangladesh; ^5^Department of Biochemistry and Molecular Biology, Shahjalal University of Science and Technology, Sylhet, Bangladesh; ^6^Department of Microbiology, University of Chittagong, Chittagong, Bangladesh; ^7^Department of Microbiology, Jagannath University, Dhaka, Bangladesh; ^8^COVID-19 Research@KTH, Department of Sustainable Development, Environmental Science and Engineering, KTH Royal Institute of Technology, Stockholm, Sweden; ^9^Department of Environmental Science and Management, North South University, Dhaka, Bangladesh

**Keywords:** COVID-19, SARS-CoV-2, revive immunity, natural diet and exercise, vitamins and minerals, antioxidants, antiviral drugs, herbal plants and medicine

## Abstract

SARS-CoV-2, a novel Corona virus strain, was first detected in Wuhan, China, in December 2019. As of December 16, 2021, almost 4,822,472 people had died and over 236,132,082 were infected with this lethal viral infection. It is believed that the human immune system is thought to play a critical role in the initial phase of infection when the viruses invade the host cells. Although some effective vaccines have already been on the market, researchers and many bio-pharmaceuticals are still working hard to develop a fully functional vaccine or more effective therapeutic agent against the COVID-19. Other efforts, in addition to functional vaccines, can help strengthen the immune system to defeat the corona virus infection. Herein, we have reviewed some of those proven measures, following which a more efficient immune system can be better prepared to fight viral infection. Among these, dietary supplements like- fresh vegetables and fruits offer a plentiful of vitamins and antioxidants, enabling to build of a healthy immune system. While the pharmacologically active components of medicinal plants directly aid in fighting against viral infection, supplementary supplements combined with a healthy diet will assist to regulate the immune system and will prevent viral infection. In addition, some personal habits, like- regular physical exercise, intermittent fasting, and adequate sleep, had also been proven to aid the immune system in becoming an efficient one. Maintaining each of these will strengthen the immune system, allowing innate immunity to become a more defensive and active antagonistic mechanism against corona-virus infection. However, because dietary treatments take longer to produce beneficial effects in adaptive maturation, personalized nutrition cannot be expected to have an immediate impact on the global outbreak.

## Introduction

Human coronavirus was first discovered in the 1960s by Tyrrell and Bynoe (1, 2), who isolated it from a young boy who was affected with the common cold and named it B814. In November 2002, a new disease appeared in the Guangdong Province of China resembling acute respiratory illness linked to pneumonia, but the actual reason was unknown (3). Later, World Health Organization (WHO) and Centres for Disease Control and Prevention (CDC) declared that a member of the Coronavirus family had never been found before in human and was the causative representative of severe acute respiratory syndrome (SARS) in April 2003. It affected 8000 people in 30 countries across five continents (4, 5). In 2004, NL63 (HCoV-NL63) Corona-virus was found in the Netherlands from a seven-month-old child with bronchiolitis and conjunctivitis (6). In 2012, Zaki et al. reported an unknown strain of Coronavirus in a patient with seven days of fever, cough, and respiratory problems in Saudi Arabia (7). Recently, a cluster of pneumonia events of unfamiliar causes was found in Wuhan in December 2019 (8, 9). SARS-CoV-2 has a mode of proliferation that allows it to spread quickly across the globe, causing mortality to a significant proportion of individuals worldwide and became a major public health concern. Although 2020 was a difficult year, 2021 appears to be even more challenging due to various SARS-CoV-2 variants. The emergence of the novel 501Y.V1 (B.1.1.7) variants of SARS-CoV-2 in the UK and 501Y.V2 (B.1.351) in South Africa was attributed to an unforeseen increase in the confirmed cases of COVID-19 in December 2020 (10). In the receptor-binding portion of the spike protein, the two variants had mutations (N501Y) that contributed 40 to 70% increased transmission (11). SARS-CoV-2 first enters into the target cell, human upper respiratory tract, by interacting with angiotensin-converting enzyme 2 (ACE2) found in epithelial cells of the respiratory tract, gastrointestinal (GI) tract and excretory system and usurp the host cellular machinery to propagate and then internalized by receptor-mediated endocytosis forming early endosome containing virus (12, 13). Following that, the virus enters into the pulmonary alveolar epithelial cells, and at acidic pH, the lipid envelope of virus fuses with the endosomal membrane (late endosome) and viral nucleic acids are released inside the host cells where viruses form negative-strand RNA (-sRNA) using the pre-existing single strand positive RNA (+ssRNA) as template and RNA polymerase enzyme (14, 15). SARS-CoV-2 composes several proteins translated into the functional RNA Polymerase protein *via* the host ribosome machinery as nucleocapsid (N), spike (S), membrane (M), and envelope (E). N protein translation occurs in the cytoplasm, whereas S, M, and E protein occur in the rough endoplasmic reticulum (RER) due to post-translational modification. The structural proteins S, M, and E combine with the viral nucleocapsid (N) (16, 17).

Various targets are presented, such as- a) The RNA-dependent RNA polymerase (RdRp) enzyme in COVID-19 is made up of the non-structural proteins nsp12, nsp8, and nsp7 (18) and acts as an effective target for preventing viral RNA production (19). b) The spike protein interacts with the ACE2 receptor to initiate the initial connection between the SARS-CoV-2 and the host (20). Other structural proteins, such as membrane M protein and envelope E protein, are important in viral entry into the host cell (21). c) Another potential target is the major protease (M^pro^), also known as viral 3-chymotrypsin-like cysteine protease (3CL^pro^), which is involved in the processing of newly translated proteins for viral replication (22, 23).

The clinical symptoms of this infection were similar to flu, a few patients developed a critical condition that included fever (87.9%), dry cough (67.71%), fatigue (38.5%), and even death (3.4%) (24–26). Additionally, myalgia or exhaustion (14.8%), sputum (33.4%), headaches (11.4%), nasal congestion (4.8%), and diarrhoea (3.7%) are typical indications and signs (24). COVID-19 Symptoms appear between 2 and14 days after infection, however, the patients might also show no symptoms; asymptomatic transmission (27). Several vaccines (e.g. Oxford-AstraZeneca, Pfizer-BioNTech, Sinopharm, Moderna, and Sputnik Janssen) have been used for COVID-19 but they are not available for all people (28–30). Moreover, several studies have already suggested the vaccine escape mutants (31). The early approval of SARS-CoV-2 vaccines has several limitations (31). It was the first mRNA/DNA vaccine without long term safety data. All of the vaccines were approved without having any prior knowledge of late side effects and duration of protection. Also, there was no authorized vaccine for human corona viruses and vaccines against common cold viruses are usually short-lived and less effective. Moreover, SARS-CoV-2 is RNA virus, so its mutation rate is so high (32). That’s why, there is no vaccine which defeated the COVID-19 disease fully (33). Because the established vaccines are made for particular strain, so the vaccine’s efficiency is low in other strains. We also don’t have the clear concept of those vaccine doses that how many doses we take or not, how many days gap between the doses and is there necessary for booster dose or not for every vaccine (34). Scientists are worked on it and try to establish a new vaccine which prevents all types of strain in COVID-19 disease (35).

Using mask while roaming outside, maintaining social distance, using sanitization, taking healthy foods, maintaining proper isolation system and always trying washing hands in every 5 minutes and avoiding touching hand or mouth (36–39). That’s the way of improving the life style that ensures strengthening the immune system even after recovery of COVID-19 infection. SARS-CoV-2 is a positive sense RNA virus; therefore, the mutation of its genome is comparatively high. That’s why no specific drug or vaccine could prevent this virus. The strengthen immunity is the only way to combat against SARS-CoV-2 and defeat COVID-19. Everybody should follow the COVID-19 protocol properly, taking healthy foods and doing physical exercise daily for boosting up the immunity to prevent and defeat this disease. A low-carbohydrate diet will help reduce the progression of diabetes, while a protein-rich diet helps keep the body in good health. It is recommended that Beta carotene, ascorbic acid, and other vital vitamins be consumed on a daily basis (40). Certain foods, such as mushrooms, tomato, and bell pepper, as well as green vegetables like broccoli and spinach, can help the body establish resistance against COVID-19 infections. The best strategy to help develop immunity is to get 7-8 hours of sleep (41). Sleep deprivation prevents the body from resting, impairing other physical activities that have a direct impact on the immune system (42).

**Figure 1:**
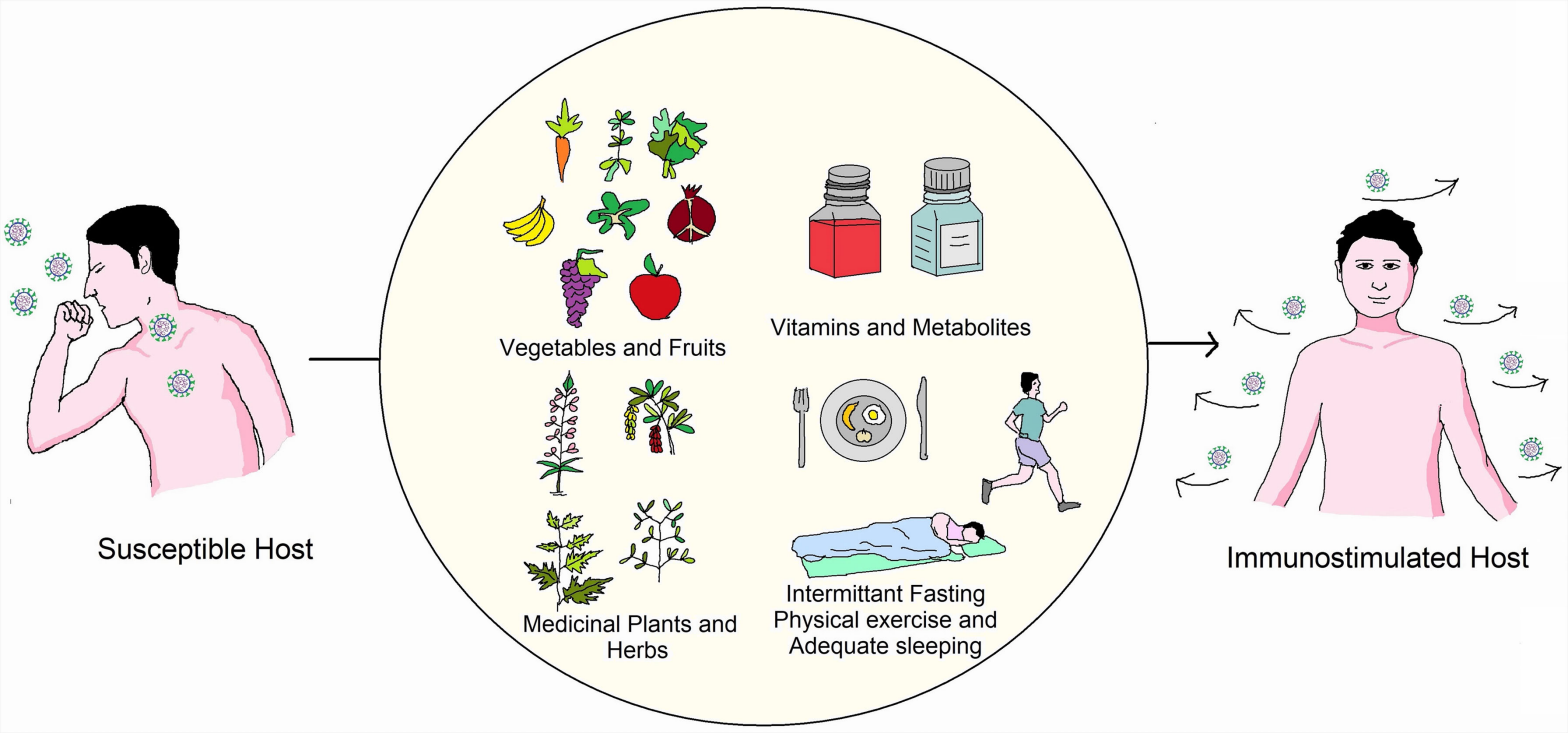
Overview of measures essential to boost immunity against COVID-19. One can boost immunity by using proper guidelines of a healthy lifestyle (Adequate food, physical exercise, sleeping).

Thus, when an individual is infected, the immune system becomes indispensable. Adaptive immunity is the system that human bodies designed to battle bacteria, viruses, and other substances that are previously unknown to them. Therefore, the immune system as a whole must be capable of recognizing and combating any infection caused by foreign substances (43). The theory demonstrates immunity, especially in young ages of individuals recovering from viral attacks. Older people and children have relatively low immunity and are thus more likely to become heavily infected with these infections (30). As the symptoms of COVID-19 are more intense in people who have pre-existing diseases and are immunocompromised, possible protection strategies include controlling pre-existing diseases and strengthening the immune system (44). As a vital regulator of the immune system, small deficiencies of some micronutrients could hamper the immune response (45). The immune system needs nutrients, including microelements and vitamins, herbal therapies, and probiotics to fight against COVID-19 (46). Additionally, dietary supplements containing minerals have a positive influence on immunological responses to viral infections.This review aims to compile all available information about strengthening the immune system to combat coronavirus infection and spread the message that prevention is far superior to cure. A schematic overview of this review has been depicted in **Figure 1**.

## Role of Immune System Against COVID-19 Infection

The immune system is the greatest defence line against any infection; it supports to human natural capacity to protect against pathogens (e.g., viruses, bacteria, fungi, protozoans, and worms) and resists infections. COVID-19 infections are undiagnosed as long as the immune system is functioning properly. Although innate immunity (quick response), adaptive immunity (delayed response), and passive immunity are the three types of defense mechanisms that work against infections, this review focuses on factors that improve innate immunity, as well as the importance of developing an effective vaccine and antiviral drugs.

The key role of the immune system against COVID-19 is precise to certain structural features of SARS-CoV-2. Particular proteins and specific sequences of the viral genome known as pathogen-associated molecular patterns (PAMPs) induce the immune response against the viruses (47, 48). Viral double-stranded RNA, nucleocapsid, membrane protein, and surface glycoprotein are some of the significant viral PAMPs. These PAMPs are accepted by specific intracellular pattern recognition receptors (PRRs) present in the human body. Upon recognizing of PAMPs, PRRs trigger the expression of certain interferon’s, cytokines, and antiviral interferon-producing genes, like- *IFN3, IFN7*, and the plethora of interleukins (IL) (49). Together, they inhibit viral replication and prevent viral spread. Contrarily, adaptive immunity works precisely and exhibits a more tailored immune response to the virus infecting the host cells. CD8+ cells and dendritic cells play the central role in this process; as they entrap, process, and present the viral antigens to the CD8+ cytotoxic T lymphocytes and natural killer (NK) cells (47, 50) followed by inducing an inflammatory response leading to the movement of monocytes and neutrophils to the virus-infected cells. These cells together with other interleukins- IL-1,6,8,21 and TNF-β kill virus-infected cells and inhibit viral replication (47, 51).

All the vital immune cells mentioned above produce cytokines and have an efficient immune response against COVID-19 that is highly dependent on the nutritional status of the host and the presence of certain vitamins and metabolites. Moreover, some external factors like- physical exercise, intermittent fasting, and adequate sleeping have also been reported to facilitate and enhance immune response (42, 52–54). Hence, the role of the immune system against COVID-19 is very vital, and it can only be replenished by maintaining some simple lifestyle practice until fully-functional vaccines and specific anti-viral drugs have been developed and reached to all the people in the world.

## Vegetables and Fruits in Daily Diet to Protect COVID-19

Vegetables and Fruits contain a plethora of vitamins, minerals, and other micro and macronutrients that help us to build up stronger resistance against viral and other infections. As there is no effective treatment for coronavirus infection, the best option is to build up immunity to prevent COVID-19 infection. For that, fruits containing vitamins and other macromolecules that can elicit potential resistance against viral infection or other respiratory tract infections should be included in the daily diet. **Table 1** enlists recommended requirements of some major nutrients that should be included in the daily diet.

**Table 1:** Recommended minimum dietary intake of essential nutrients for adults.

Essential Nutrient	Amount		References
	Male	Female	
**Carbohydrates**	130 g/day	130 g/day	(55)
**Protein**	56 g/day	46 g/day	(55)
**Vitamins**	0.131 g/day	0.114 g/day	(56)
**Water**	2.5 lit./day	2 lit./day	(57)

Carrots, kale, mangoes, spinach, apricots, broccoli, sweet potatoes, squash, and cantaloupe contain a good amount of vitamin A that can be added to the daily diet. The vegetables mentioned above and other colorful vegetables contain beta carotene, which when consumed, gets converted into vitamin A considered crucial for a robust immune system. Many studies support that vitamin A has both promoting and regulatory roles in both innate and adaptive immunity (58). Therefore, it can enhance the immune system and provide improved defense against coronavirus infection.

It is known that Acute Respiratory Distress Syndrome (ARDS) is a crucial factor of fatality in SARS-CoV-2, rapid release of high amounts of free radicals and increased oxidative stress leads to cellular catastrophe, internal organ failure, and eventually death. Lentils, beans, tofu, chickpeas, fortified cereals, seeds, nuts, wheat germ, etc., should also be considered as an important food component. These plant-derived products are rich in zinc, which enhances immunity toward viral infection by altering the resistance of the host (59, 60).

## Medicinal Plants and Herbs to Reinforce Protection Against Viral Infection

Various plants, herbs, or their specific parts are traditionally used worldwide due to their antimicrobial activity. Many antimicrobial agents extracted from different plants can promote immune cell proliferation and enhance the immune response by providing an excellent antiviral activity. Anti-inflammatory medicinal plants may have a pleiotropic role in COVID-19 treatment, as elevated levels of inflammatory markers such as interleukin (IL)-6, erythrocyte sedimentation rate (ESR), and C-reactive protein (CRP) have been linked to severe disease and poor outcomes in COVID-19 patients, most likely due to cytokine storm (61).

Garlic has antiviral activity against herpes simplex virus 1 (HSV-1), influenza A and B, viral pneumonia, human immunodeficiency viruses (HIV), and common cold-causing rhinoviruses (62). Recent research also shows that garlic can enhance immunity by activating protective immune cells against viral infection (63). Garlic appears to boost immune system activity by modulating cytokine production, phagocytosis, macrophage activation, and immunoglobulin synthesis by activating NK cells, lymphocytes, eosinophils, and dendritic cells (DC) (64).

Ginger due to the high concentration of potent plant compounds, has been a potential antiviral agent for centuries. *In vitro* research has shown that the extract of ginger has some antiviral activities on avian as well as respiratory syncytial viruses (RSVs) (65, 66). In addition, gingerols and zingerones impede viral replication and counteract the viruses from penetrating host cells (67). The ginger extract inhibited TNF-α, IL-12, IL-1β (pro-inflammatory cytokines) production in LPS stimulated macrophages. Ginger extract down-regulated the expression of B7.1, and MHC class II molecules. Researchers studied the effect of ginger extract on the expression of proinflammatory cytokines and chemokines in murine peritoneal macrophages and also observed a reduction in T cell proliferation at a significant level in response to all stimulation (68).

Oregano is well known for its medicinal properties, a common herb in the mint family. The prime compound found in it is carvacrol, which provides antiviral properties. Oregano oil and carvacrol exhibited antiviral activity against herpes simplex virus (HSV), rotavirus, and, importantly, respiratory syncytial viruses (RSVs), causing respiratory infection (69, 70).

Tulsi is the holy variety of basil with the property to increase immunity to help us against viral infection. A study showed that extract supplementation of holy basil notably raised T cells levels and NK cells (71).

Basil includes compounds such as apigenin and ursolic acid that have potent antiviral activity in opposition to many viral infections in a particularly sweet variety of basil (72). It has also been found to boost up the immunity that may assist in striving for other diseases.

Sage a member of the mint tribe, is a scented herb. It has been utilized from age-long to medicate viral type infections in conventional medicine (73). Safficinolide and sage, contained in the plant leaves and stems, are the compounds responsible for the antiviral properties (74). One study by Geuenich et al. reported significant inhibition of HIV entry into the host cell following the application of sage extract, which is also a RNA virus-like SARS-CoV-2 (75). Further study may establish potential preventive measures against COVID-19.

Fennel is a licorice plant with a potent antiviral effect on herpes viruses and parainfluenza type-3, which cause cattle breathing infections (76). The key element of fennel essential oil, trans-anethole, has demonstrated promising antiviral effects in opposition to the herpes virus (77). It can also improve the immune system and relieve inflammation, according to animal-based studies (78).

Lemonbalm is commonly used in tea and seasoning as a lemony herb, but it has some medicinal significance as well. Lemon balm extract is a potent essential oil that has antiviral activity, and a study has already shown it has significant antiviral effects against HIV-1 (75), avian influenza (79), herpesviruses (80, 81) as well as enterovirus (82).

Licorice is one of those herbs that has been used in traditional Chinese medicine for years (83) Some of the active substances in licorice are glycyrrhizin, liquiritigenin, and glabridin, which exhibit significant antiviral properties (84). The root extract of licorice has been found effective against Influenzae (85), HIV, RSV (86), HBV (83), herpes virus (87), and, most importantly, SARS-CoV-2 (88).

Dandelion although considered as a weed has been studied for possible antiviral effects. Research has shown that replication of hepatitis B (89), HIV (90), and influenza (91) can be blocked by dandelion.

Moringa leaves and seeds are enriched in vitamin C, vitamin A, calcium, and potassium content. The traditional, industrial, nutritional, and medicinal values used in folk medicine are diversified in *Moringa oleifera* for numerous health reasons, especially for the symptoms (e.g., fever, muscle pain, or asthma) of COVID-19 patients (92). *Moringa peregrina* has several pharmacological properties, including anti-microbial, anti-diabetic, antioxidant, anti-inflammatory, anti-spasmodic, anticancer, reduction of lipid activity, and cognitive problems (95). Black cumin, a component of this plant, is well-known in obstructive respiratory conditions for its robust immune regulation, anti-inflammation, and antioxidant advantages that have been used for decades for medicinal purposes (93, 94). Thymoquinone, Nigellidine, and α-hederine from *N. sativa* can impact the immune responses against COVID-19 on molecular grounds (95, 96).

Plants including *Glycyrrhiza glabra*, *Allium sativum*, and *Clerodendruminerme Gaertn* have been proven to impede SARS-CoV virus replication, making them potential SARS-CoV-2 fighters (97). In India, extracts of sunthi (*Zingiber officinale* Roscoe.), lavanga (*Syzygiumaromaticum*), and maricha (*Piper nigrum*) are used to prevent and treat COVID-19 because they stimulate humoral and cell-mediated responses and reduce respiratory hyperresponsiveness and nasal congestion (98). *Andrographis paniculata*, a tropical species found in South Asia, inhibited elevated NOD-like receptor protein 3 (NLRP3), caspase-1, and IL-1 molecules, all of which are associated in the pathogenesis of SARS-COV and, most certainly, SARS-CoV-2 (97). Decoction of *Tinospora cordifolia*, *Andrograhispaniculata*, *Cydonia oblonga*, *Zizyphus jujube*, *Cordia myxa* and *Arsenicum album* 30 have been suggested to prevent respiratory infections (97, 98). In China, Lianhuaqingwen, a Traditional Chinese Medicine (TCM) formula comprised of 13 herbs (**Supplementary Table 1**), is being used as a possible therapeutic candidate for the management and cure of COVID-19 because it inhibited SARS-CoV-2 replication, lowered pro-inflammatory cytokine secretion, and altered the morphology of SARS-CoV-2 cells (99, 100). Furthermore, a study report demonstrated the bioactive efficacy of extracts *Sanctellaria baicalensis* containing baicalin, which is considered one of the most important TCM herbal constituents, as well as hesperetin, an active component found in tangerine peel, in alleviating COVID-19 symptoms (98, 101). A range of investigations are being carried out in order to develop a formulation against coronavirus using medicinal plants and herbs (**Table 2**). The biomolecules are the basis for establishing successful treatment against several protein targets of SARS-CoV-2 including Nsp1, Nsp3 (Nsp3b, Nsp3c, PL^pro^ and Nsp3e), Nsp7-Nsp8, Nsp9-Nsp10, Nsp14-Nsp16 complexes, 3CL^pro^, E protein, ORF7a, Spike (S) glycoprotein, C-terminal RNA binding domain (CRBD), N- terminal RNA binding domain (NRBD), helicase and RdRp (115). Despite the fact that various medicinal plants have been identified as efficient antiviral treatments for COVID-19, additional research is required.

**Table 2:** Applications of medicinal plants and herbs as possible therapeutics against COVID-19 infection.

Scientific name	Active compound	Mode of action	Reference
***Cannabis sativa***	Cannabinoid cannabidiol	Anti-inflammatory effect on basis of modulating the gene expression of ACE2 enzyme, serine protease TMPRSS2, protein required for SARS-CoV-2 entrance into host cells.	(102)
***Glycyrrhiza glabra***	Glycyrrhizin, glycyrrhetic acid, liquiritin and isoliquiritin	Counterbalance the activity of COVID-19.	(103)
***Citrus* sp.**	Essential oils, pectin, naringin and hesperidin	Binds to high SARS-CoV-2 cellular receptors affinity with strong affinity, limiting the proinflammatory response of immune system.	(104)
***Porphyridium* sp.**	Carrageenan (Sulfated polysaccharides)	Coronavirus inhibitors with high potency that impede virus attachment or uptake into host cells.	(105)
***NilavembuKudineer***	Benzene 123 Triol	Immunoregulatory action against the ACE2 enzyme receptor, which facilitates viral entrance during SARS-CoV-2 pathogenesis.	(106)
***Nigella sativa***	Nigelledine, α- Hederin	Inhibitory action of Proteases inhibiting activity; active sites of CoVs (3CL^pro^/M^pro^) (PDB ID6LU7 and 2GTB).	(107)
***Camellia sinensis***	Polyphenols (Sanguiin, The aflavin gallate, The aflavin digallate, Kaempferol, Punicalagin and Protocatechuic acid)	COVID-19 protease (M^pro^) inhibitor, inhibits viral replication inside the host	(108)
***Zingiber officinale***	6-gingerol	Stronger binding affinity at active sites of R7Y COVID-19, the major protease required for growth and multiplication of SARS-CoV-2	(109)
***Scutellariabaicalensis***	Baicalein	Anti-SARS-CoV-2 efficacy by inhibiting SARS-CoV-2 3CL^pro^ and multiplication.	(110)
***Allium sativum***	Essential oil	Served as ACE2 receptor antagonist for resistance against Coronavirus as well as SARS-CoV-2 main proteases inhibitor	(111)
***Ginkgo biloba***	Ginkgolide A, Terpenoids	Stronger link and binding ability with proteases.	(112)
***Camellia sinensis***	Epigallocatechin gallate	COVID-19 major proteases, 2019-nCoV S2 subunit post fusion core, prefusion spike glycoproteins, and SARS-CoV-2 NSP15 endoribonuclease are all targets.	(113)
***Curcuma longa***	Hesperidin, Rutin, Diosmin, Apiin,Diacetyl curcumin	Inhibition of the major SARS-CoV-2 protease (M^pro^).	(110)
***Eucalyptus* sp.**	Jensenone	COVID-19 major protease (M^pro^) inhibitor	(114)
***Andrographispaniculata* sp.**	Flavonoids, 14-deoxy-11,12- didehydroandrographolide	Inhibition of NLRP3, caspase-1, IL-1ß, RdRp	(115)
***Cynara scolymus***	Rhoifolin, flavonoids	Inhibition of S protein, major protease (M^pro^) and ACE2	(115)
***Rhus succedanea***	Rhoifolin	Inhibition of S protein, major protease (M^pro^)	(115)
***Swertiapseudochinensis***	1,7-dihydroxy-3-methoxyxanthone	Inhibition of SARS-CoV-2 RdRp	(115)
***Matricaria chamomilla***	Apigenin (Flavone)	Blocks the proteolytic activity of SARS-CoV-2 3CL^pro^.	(116)
***Gnidialamprantha***	Gnidicin, gniditrin (Diterpene esters)	Inhibit SARS-CoV-2 RdRp.	(116)
***Pueraria lobata***	Puerarin (Iso-flavone)	Anti-SARS-Cov 3CL^pro^ enzyme activity.	(116)

ACE2, Angiotensin-Converting Enzyme 2; TMPRSS2, Transmembrane protease; serine 2; SARS-CoV, Severe acute respiratory syndrome coronavirus; COVID-19, Coronavirus disease-19; 3CL^pro^, 3 Chymotrypsin-like proteases; M^pro^, Main protease; NSP15, Nonstructural protein 15; NLRP3, NOD-like receptor protein 3; IL-1ß, Interleukin-1ß; RdRp, RNA-dependent RNA polymerase.

## Vitamins and Food Supplements to Enhance Immunity Against Viral Infection

Vitamins significantly boost immunity by supporting different biochemical reactions important for immune response and helping fight infections. Hence, vitamins are considered to be a potential weapon to prevent coronavirus infection (117). A comprehensive table of vitamin requirements for different age groups is given in **Table 3**.

**Table 3:** Daily Requirement of Vitamins for Different Aged People (118).

Category	Age	Vitamin C (mg)	Vitamin D (µg)	Vitamin K (µg)
Males	11-14 years	50	10	45
	15-18	60	10	65
	19-24	60	10	70
	25-50	60	5	80
	51+	60	5	80
Females	11-14 years	50	10	45
	15-18	60	10	55
	19-24	60	10	60
	25-50	60	5	65
	51+	60	5	65

mg, Milligram; µg, Microgram.

Vitamin A are available as its active forms retinal, retinol, and, especially, retinoic acid (RA), which mediate the transcription of hundreds of genes implicated in several biological pathways (119). Vitamin A enhanced the antioxygenic capacity of the tissues in extended amounts and it is proposed that retinol could even be regarded as an antioxidant similar to the tocopherol in human nutrition as well as play a key role in the modulation and regulation of the immune system, including innate and adaptive responses, which may enact a crucial element in the fight against pathogens (119, 120). A prospective, multicenter observational cross-sectional study showed that vitamin A plasma levels in COVID-19 patients are reduced during acute inflammation and that severely reduced plasma levels of vitamin A are significantly associated with ARDS and mortality (120). Systematic reviews and meta-analyses have reported that retinoid administration improves symptoms related to acute pneumonia, and also reduces incidence, morbidity, and mortality of measles (119). Therefore, and even though there is still no clinical evidence regarding vitamin A and COVID-19, in the light of its roles in lung function and immunity, the vitamin is currently being investigated for the treatment of SARS-CoV-2 infection alongside with other antioxidants.

Vitamin B performs a significant role in the body’s immune system. Since the lack of vitamin B will impair the immune reaction of the host, patients diagnosed with the virus should be supplemented to strengthen their immune system (87). Some of the food products rich in folate (natural form vitamin B) - are the kernel, peanuts, fruits, and green vegetables (121).

**Figure 2:**
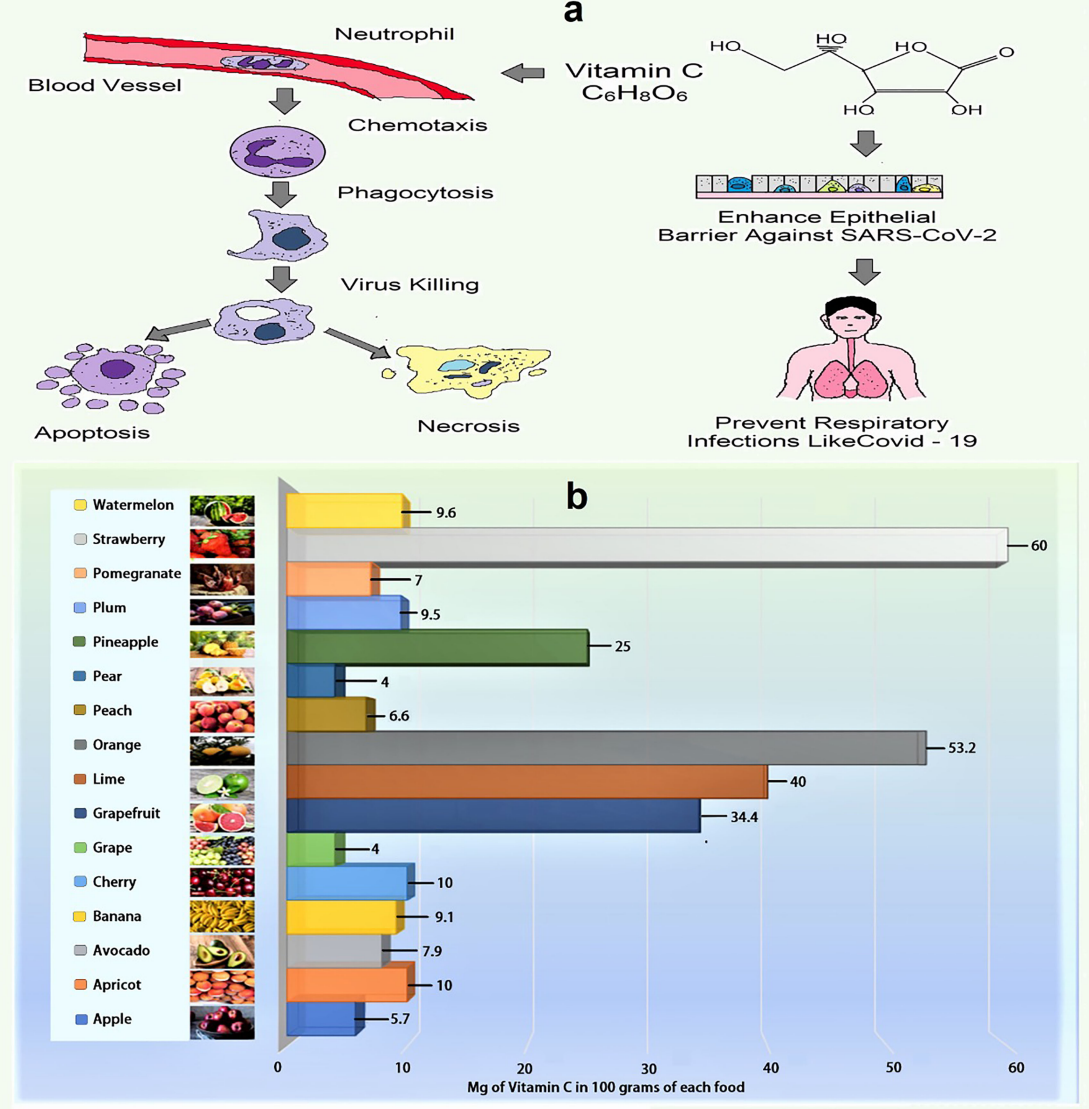
**(A)** Role of vitamin C in phagocytosis (Vitamin C promotes neutrophil movement in response to chemoattractants and microbes engulfment, as well as formation of reactive oxygen species and microbial death. It also stimulates caspase-dependent apoptosis, boosting macrophage uptake and clearance, and suppresses necrosis, including NETosis, promoting inflammatory resolution and reducing tissue damage). **(B)** Amount of vitamin C in selected fruits (per 100 g) (124–126).

Vitamin C is a water-soluble vitamin that acts as an effective reducing agent, as well as having antioxidant, immunomodulating, antiviral and antithrombotic activities. It plays an important role in the immune system by supporting the epithelial barrier against pathogen invasion and the cellular processes of the innate and adaptive immune systems (117, 119, 122, 123). **Figure 2** summarizes the role of vitamin C in phagocytic cells that participate in pathogen defense and the amount of vitamin C found in certain common fruits (119, 127). Vitamin C may boost the immune response to viral infections by stimulating T-lymphocytes and NK-lymphocytes proliferation and activation, as well as interferon production (124). It also increases the quantity of antibodies in the blood and aids lymphocytes differentiation, both of which are necessary for resistance to viral infection (110). Vitamin C strengthens the immune system (**Supplementary Table 2**) and protects against coronavirus infection (128). For example, it enhanced the resistance of chick embryo tracheal organ cultures to infection and protected broiler chicks against an avian coronavirus as well as can ameliorate flu-like symptoms (sneezing, a running nose, and swollen sinuses) as a mild antihistaminic agent (124, 129, 130). Furthermore, vitamin C therapy reduced the effects of sepsis on pulmonary dysfunction in septic mice with ARDS by down regulating proinflammatory genes and improving epithelial membrane permeability (124, 125). A recent report revealed that, vitamin C increases the antiviral activity of lung epithelial cells and protects lung fibrosis and damage in both *in vivo* and *in vitro* models (131). Previous placebo-control trials found that high-dose vitamin C (2-16 g/day) was successful in reducing the mortality of patients with sepsis, severe influenza, and acute lung damage as well as reducing the length of stay in the intensive care unit (ICU) and the duration of mechanical ventilation (124, 125, 131). Additionally, vitamin C may modulate the cytokine storm, which is characterized by elevated levels of the proinflammatory cytokine interleukin (IL)-6, increasing the likelihood of respiratory failure necessitating mechanical ventilation in COVID-19 patients (125). As severe COVID-19 infection can lead to sepsis and ARDS, numerous studies have attempted to alleviate these symptoms with high doses of vitamin C (131). Several clinical trials have been ongoing since initial trials in China and the United States reported the beneficial effects of high-dose intravenous (IV) vitamin C, but due to the excellent safety profile of vitamin C and the need for ICU treatment for a huge percentage of COVID-19 cases, clinical application of vitamin C has been proposed by many authors, even before the outcomes of large clinical trials are available (119, 124, 131).

**Figure 3:**
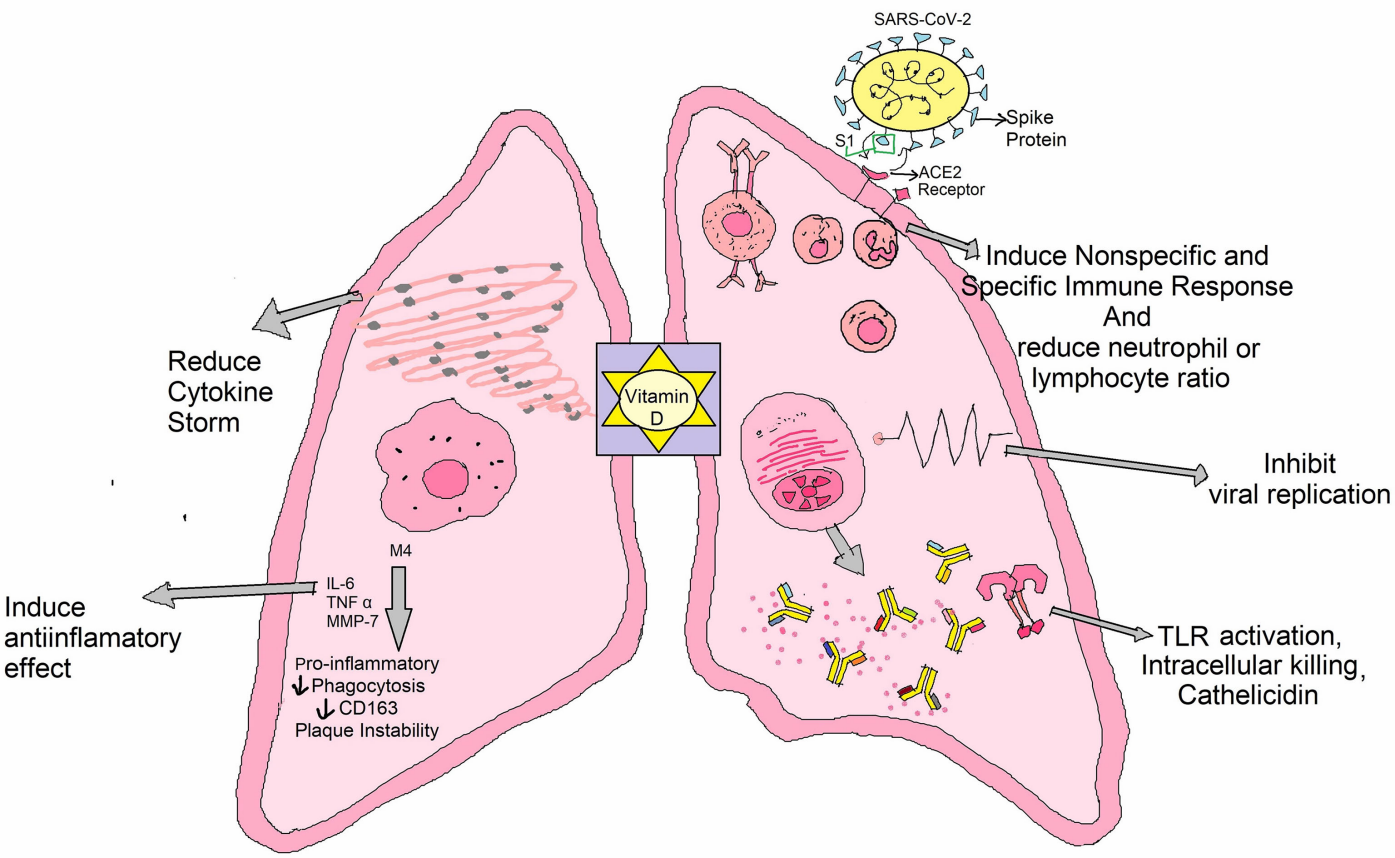
Role of vitamin D against COVID-19: It has an impact on SARS-CoV-2 infection outcomes through a variety of mechanisms, such as inducing anti-inflammatory effects and/or lowering neutrophil/lymphocyte ratio, provoking innate and acquired anti-viral responses, interacting with cellular factor ACE2, conversing with viral aspects, and/or disrupting virus life span.

Vitamin D is a type of fat-soluble secosteroid responsible for enhancing the intestinal synthesis of calcium, magnesium, phosphate, and many other biological activities (**Supplementary Table 2**) (132). In humans, vitamin D2 (known as ergocalciferol) and vitamin D3 (cholecalciferol) are the most common compounds from this class of vitamin (133). It usually acts to stimulate the endogenous and dampen the adaptive immune systems (**Figure 3**) (134). The risk of acute respiratory tract infections and asthma exacerbation can be marginally mitigated through vitamin D supplementation (135). It can be synthesized with the support of sunlight in the bodies (136). The vitamin D receptor (VDR) is also located in lung epithelial cells, where it regulates the synthesis of defensins and catelicidins, peptides that have antiviral effect either directly or through immunological regulation (125). The decreased vitamin D level in the calves was documented to raise susceptibility to bovine coronavirus infection (132, 137). It has been hypothesized that the reduced antiviral immune response in COVID-19 patients during vitamin D deficiency could be attributable to a decrease in LL37 levels, an antimicrobial peptide generated from catelicidin (125). Vitamin D might help to reduce the severity of inflammatory reactions by inhibiting pro-inflammatory cytokines (TNF-α and IL-6) which are associated in the development of cytokine storm in COVID-19 related ARDS (125, 137). In addition to its immune function, vitamin D, like Zn and vitamin C, is vital in the development and maintenance of epithelial and endothelial boundaries, including lung cell (125). According to several studies, there is a link between vitamin D insufficiency and SARS-CoV-2 infection susceptibility and clinical manifestations (125, 137). As a result, persons living in isolation need often subject themselves to direct sunlight and refill their dietary requirement of foods rich in vitamin D along with intake of vitamin D tablets commonly known as D-Rise tablet upon consultation of physicians, to maintain immune function. More clinical research is required to assess the effects of vitamin D supplementation in COVID-19 patients.

Vitamin K is used for its cardiovascular health role, but scientists have revealed that it may also be advantageous for COVID-19 therapy. A new analysis by a team from the Bispebjerg Hospital in Denmark found that COVID-19 patients have low vitamin K levels and its deficiency is one variable that may imply higher death in patients (138). An investigation by Canisius Wilhelmina Hospital, researchers in the Netherlands, found that optimal levels of vitamin K play a pivotal role in blood clots and pulmonary dissuasion (139). These findings indicate that increased vitamin K intake may aid patients to combat COVID-19 better (93). Animal and plant sources for various vitamins are enlisted **Supplementary Table 3**.

Importantly, Vitamin A deficiency can cause osteoporosis if consumed in large amounts. This can increase the risk of fractures, especially in people who have already been diagnosed with osteoporosis (140). The high dose of vitamin E intake increasing lung cancer (141). The high dose of vitamin B absorbed increasing the growth of cancer cell (142). The high level of vitamin D is lower risk of breast cancer because it increased 25-hydroxyvitamin D (25-OH D) (143, 144).

There is no proven preventive technique to prevent the deadly COVID-19, a global issue where hydroxychloroquine (HCQ) was initially employed to counteract the COVID-19 viral load (145). Both the CQ and HCQ act as an immunomodulator. The clinical studies have found that in the COVID-19 patients, the increased concentration of cytokines in the plasma was observed (146). Remdesivir, an antiviral medication, prevented the virus from multiplying and was created to combat Ebola. The usage of this medicine aided the early recovery of the afflicted person. Because no medication has yet been discovered to be particularly successful against COVID-19, some antiviral drugs have been used in clinical research to treat COVID-19 patients (147, 148). Several drugs, including ribavirin, favipiravir, femdesivir, tenofovir, baricitinib, oseltamivir etc. are in trial phase (149, 150).

## Limitation of Drugs That Used to Treat the COVID-19 Infection

There are currently no particular COVID-19 medications available however, several authorized drugs and novel antiviral compounds have been repurposed as prospective antiviral choices for COVID-19 infection treatment (151, 152). As a result, various pharmacological classes are being repurposed, including antivirals (like indinavir, ritonavir, saquinavir, darunavir, lopinavir, favipiravir, remdesivir, galidesivir, emtricitabin, tenofovir, oseltamivir, penciclovir, ganciclovir, ribavirin, umifenovir etc.), immunomodulators (like tocilizumab and interferon), enzyme inhibitors (like nafamostat, camostat and carfilzomib), anti-malarial (like chloroquine and hydroxychloroquine), anti-parasitic (ivermectin), anthelmintics (niclosamide), antibiotics (like teicoplanin, azithromycin,eravacycline, valrubicin, streptomycin, nitazoxanide, caspofungin, and colistin), anti-rheumatoid (like baricitinib), corticosteroids and herbal medicines (151–155). There are also several obstacles to overcome in terms of dose adjustments, acute/chronic toxicity, and unfavorable *in vivo* pharmacokinetic (PK) properties (e.g. plasma protein binding, tissue distribution, drug interactions), as well as the selection of an appropriate delivery system and method of exposure to deliver repurposed drugs against COVID-19 (151, 154). Another drawback of COVID-19 drug is that their proclivity for causing acute toxicity may surpass the uncertain benefit of a specific antiviral therapy (156). For example, the premature use of hydroxychloroquine in COVID-19 treatments may result severe skin lesions, liver failure, hypoglycemia, cardiovascular disorder, gastrointestinal upset, neuropsychiatric effects, and retinopathy, as well as an inferred significant threat of QT prolongation and ventricular arrhythmia in the case of its combination with azithromycin (153, 156–158). Antiviral drugs like lopinavir/ritonavir/remdesivir/favipiravir have been linked to nausea, diarrhea, hepatotoxicity, vomiting, gastroparesis, or rectal bleeding, abnormal liver function, renal impairment, higher serum uric acid, hypotension, and rashes, whereas ribavirin, also known as a teratogen and contraindicated in pregnancy causes severe dose-dependent hematologic toxicity including hemolyticanemia (61%), hypocalcemia (58%), and hypomagnesemia (46%) (156, 158, 159). Other drugs that have been reported to have side effects include fever, myalgias, headaches, leukopenia, lymphopenia, autoimmune hepatitis, and thyroid disease for interferon, upper respiratory tract infection, malignancy, thrombosis, nausea, blurred vision, hypocholesterolemia, hypertension, urinary tract infection and skin cancer for baricitinib,and are nausea, rashes, dizziness, fever and tachycardia for ivermectin (158, 159). Likewise, many other drugs are also linked to a variety of side effects, and further research in needed to confirm their therapeutic usefulness (153). In immune-compromised individuals, the emergence of viral resistance, viral dormancy, and recurrent infection is a downside of antimalarial drugs, whereas oral dosing of niclosamide is a limitation in its capacity to reach the infection site due to poor absorption (97, 154). The use of corticosteroids is still debated, and current World Health Organization guidelines advise against using them unless there is some concurrent indication, such as chronic obstructive pulmonary disease exacerbation or pressor-refractory shock (156). Some of the drawbacks of medicinal plants include a lack of information on the safety profile and dosage for various ailments (155). There are still no well-designed PK and pharmacokinetic-pharmacodynamic (PK-PD) data of COVID-19 patients available, as well as no high-quality evidence to support the use of the above repurposed medications for COVID-19 treatment. As a result, well-designed PK and PD studies particularly targeting COVID-19 patients are immediately needed to improve our understanding of the repurposed medicines’ dose-exposure-effect correlations (151).

## Food Supplements Play Important Role in Immunity Boost Up

**Probiotics** prevent respiratory tract infections like coronavirus infection, a research study showed that supplementation with *Bifidobacterium breve YIT4064* augments the antibody response against oral influenza virus and producesa significantly greater amount of high serum anti-influenza virus-specific IgG (160). Supplementation with *Lactobacillus casei* also conferred significantly increased protection to the upper respiratory infection by intra-nasal influenza virus (161). Another study performed on children aged 1-6 years, who were fed with milk supplemented with *Lactobacillus rhamnosus* showed a lower incidence of respiratory tract infections compared to the control group (162).

Honey, another widely used food supplement potentiate to augment the innate immune system and may help fight against COVID-19 (163). It has been found to cause cell division (mitosis) in both B- and T-cells (164). Because it can activate T-lymphocytes, B-lymphocytes, and neutrophils, leading to the production of cytokines. Therefore, it plays a crucial role in generating an adaptive immune response to SARS-CoV-2 (165). Sugars, organic acids, amino acids, phenolic compounds, vitamins, and minerals are among the components found in honey. This is why honey has been researched in animal and human models for a long time to determine its antioxidant potency (165–167). Honey has also been shown to reduce acute respiratory distress symptoms when consumed daily (168).

Zinc (Zn), is an essential trace element, also known as the “magic bullet,” that is required for the normal human cell activity, the protection of normal tissue barriers including the respiratory epithelium, inhibiting pathogen invasion, and a healthy immune and redox systems (169). Its potent immunoregulatory and antiviral properties, as well as its importance in growth, reproduction, immunity, and neurobehavioral development, have made this mineral and its ionophores potential COVID-19 targets (59, 125, 170). Zn has the ability to increase T cell growth and activity, so decreasing the cytokine storm, which is characterized by high amounts of proinflammatory cytokines and chemokines that cause systemic immune response dysfunction, resulting in ARDS or multiple organ failure (125). It also contributes to the integrity of epithelial barriers, which are necessary for organism defense and pathogen invasion prevention (59, 125). Several studies have looked into the role of Zn in immunity and host susceptibility and its insufficiency is most likely one of the variables that predisposes people to infection and advancement of COVID-19 (169, 171). *In vitro* tests found that the Zn ionophore pyrrolidine dithiocarbamate inhibited influenza virus replication, and that modest doses of Zn and Zn-pyrithione limit SARS-CoV-1 replication by inhibiting RNA polymerase, which is required for RNA virus replication (59, 125, 137, 172, 173). Zn can also improve interferon (IFN) cytokine signalling against RNA viruses and reduce angiotensin-converting enzyme 2 (ACE2) activity, which is required for SARS-CoV-2 entrance into host cells (125). Zn, as a result, suppresses the elongation phase of RNA transcription. Further, it may boost antiviral immunity by increasing IFNα levels in leukocytes *via* JAK/STAT1 signaling pathway (173). A recent *in vitro* study found that low zinc levels promote viral growth in SARS-CoV-2 infected cells while, in a complete metal(loid)s investigation, the amount of Zn, Mg, manganese, iron, lead, arsenic, and thallium was lower in severe COVID-19 patients compared to non-COVID-19 patients (174, 175). Consequently, control trial and meta-analyzed data revealed that a significant number of COVID-19 patients were Zn deficient (88, 175), which was associated with a longer hospital stay and increased mortality, whereas, Zn treatment has been reported to reduce the severity (up to 54%) and duration of various cold symptoms, such as fever, cough, sore throat, muscle pain and nasal congestion (88),which can occur after SARS-CoV-2 infection as well as decreased pneumonia in infants, and decreased mortality (59, 123). Although it is unknown whether Zn supplementation can help patients with lower respiratory tract infections, because of its direct antiviral activity, it may alleviate tissue injury caused by mechanical ventilation in COVID-19 patients who are critically ill with low Zn level, and it may be used in conjunction with antiviral drugs to treat COVID-19 infection (169, 173). Zn is now being explored for prophylaxis and treatment of COVID-19 patients due to its role in immune function and ability to limit coronavirus replication. The usual dose for Zn varies (2-13 mg/day), irrespective of age and sex, with the upper safety threshold for Zn is set at 40 mg/day by the Institute of Medicine (IOM), and it is worth emphasizing that prior to taking Zn, physicians’ advice should be asked to mitigate deleterious consequences (175).With more investigation, it might provide a cost-effective therapy for COVID-19, which is obviously needed in this pandemic.

Magnesium (Mg) as an enzymatic activator that plays a role in a variety of physiological activities including cell cycle, metabolic regulation, muscle contraction, and vasomotor tone, as well as anti-inflammation, anti-oxidation, immunological response, anti-spasm, vasodilation, and neuroprotection (176, 177). Mg is a necessary cofactor for oxidative phosphorylation, energy production, protein synthesis, glycolysis, and nucleic acid synthesis and maintenance, among other biological activities (approximately 600) (178, 179). It functions as a structural or catalytic component on enzymes as well as on substrates and it is required for oxidative phosphorylation, energy production, protein synthesis, glycolysis, and nucleic acid synthesis and maintenance (178). As a result, it significantly supports the hypothesis that magnesium deficiency may influence SARS-CoV-2 susceptibility and response. Mg sulfate, a calcium antagonist, is often utilized as a bronchodilator in the adjuvant treatment of asthmatic patients (178). By blocking the IL-6, NF-kB, and L-type calcium channels, it also reduces inflammatory responses and oxidative stress, as well as improving lung inflammation (178). Accordingly, Mg sulfate has a promising aspect in the treatment of pulmonary symptoms, with the potential to reduce respiratory distress and enhance lung function in COVID-19 patients. There is insufficient information on the magnesium status of people with Covid-19 of various severity levels. According to a retrospective study, lower Mg levels were seen in patients with more severe COVID-19 symptoms (61). In a recent transversal investigation, hypomagnesemia was found to be common in hospitalized Covid-19 patients, but high-level serum magnesium concentration was more frequent in critical condition (176). Surprisingly, Mg deficiency has been recorded in up to 60% of critically ICU patients, predisposing these patients to serious, even life-threatening effects such as hypokalemia and hypocalcemia (178). According to a cohort research, the combination oral treatment of Mg (150 mg daily), vitamin D (1000 IU daily), and vitamin B12 (500 mcg daily) considerably decreases the proportion of older COVID-19 patients who need oxygen and/or critical care support (180). In fact, Mg sulfate prolonged infusion could be used as a supplement to other treatments for COVID-19-infected severely ill individuals. Mg and vitamin D are required for the immunological function and cellular resilience in a variety of organs. In particular, Mg aids in the synthesis of vitamin D, which helps to control calcium and phosphate equilibrium to impact bone formation and maintenance (61, 178). A lack of either these nutrients has been linked to a variety of ailments, including skeletal abnormalities, cardiac diseases, and metabolic disorders, as well as the cytokine storm seen in the COVID-19 infection (179, 181). In a group of COVID-19 patients, oral therapy with Mg, vitamin D, and vitamin B12 reduced the need for oxygen assistance, intensive care admission, or both (180). Hence, it is prudent to address not only vitamin D deficiency but also Mg deficiency and in COVID-19 patients, the prescribed dose of Mg (**Supplementary Table 4**) need to be taken in order to achieve the most advantages of Vitamin D (178, 179). Of obviously, given the unique circumstances surrounding the COVID-19 epidemic, extensive clinical data is necessary in future study to determine whether Mg sulfate in combination with other approved therapeutic medicines is more useful to COVID-19 patients’ health.

Copper’s role in immunity and host susceptibility to infection is proved in several studies (24, 182, 183). A recent study at the University of Southampton has revealed that copper can successfully inhibit the proliferation of respiratory viruses, such as those associated with SARS and MERS outbreaks (184, 185). It would be worth exploring the implementation of the same strategy against COVID-19. The absence of copper in the body leads to lower IL-2 proliferation and also lowers the potential to generate superoxide anion and destroy microorganisms that have been ingested (24). Some food can be used as a source of copper as oysters, nuts, seeds, chicken marinated mushrooms, lobster, liver, leafy greens, and dark chocolate (121). It enhances the reaction of T lymphocytes, such as replication and IL-2 secretion (186).

Selenium is yet another micronutrient that has an important role to play when it comes to maintaining human health. Selenium impacts all the immune responses– innate, non-adaptive, and adaptive (187). Food rich in selenium includes beef, turkey, chicken, seafood, shellfish, and eggs (121). It enhances immune cell production against various pathogens (187–189). The low levels of selenium decrease the activity of the natural killer cell and increased mycobacterial disease (189, 190). It can improve various aspects of human immune function (100 - 300 μg/day), including older adults (191).

Beta-glucans contribution in promoting stimulation against viral attack has been demonstrated in numerous human trials (192, 193). Results of several studies have shown a reduction in cold and flu symptoms and upper respiratory infections when compared to placebo. The mechanism of action of beta-glucans against viruses other than COVID-19 might be promoting viral elimination or inhibition by priming innate immune function (194). Recent studies show the possibility of both a prophylactic and a curative advantage of sulforaphane against ARDS and SARS-CoV-2 (195, 196).

The supplements mentioned above may have immune-boosting properties, according to the outcome of scientific studies. However, several of these supplements have not been extensively tested in humans, emphasizing the need for future research.

## Immunity-Boosting Properties of Different Metabolites Against COVID-19

**Figure 4:**
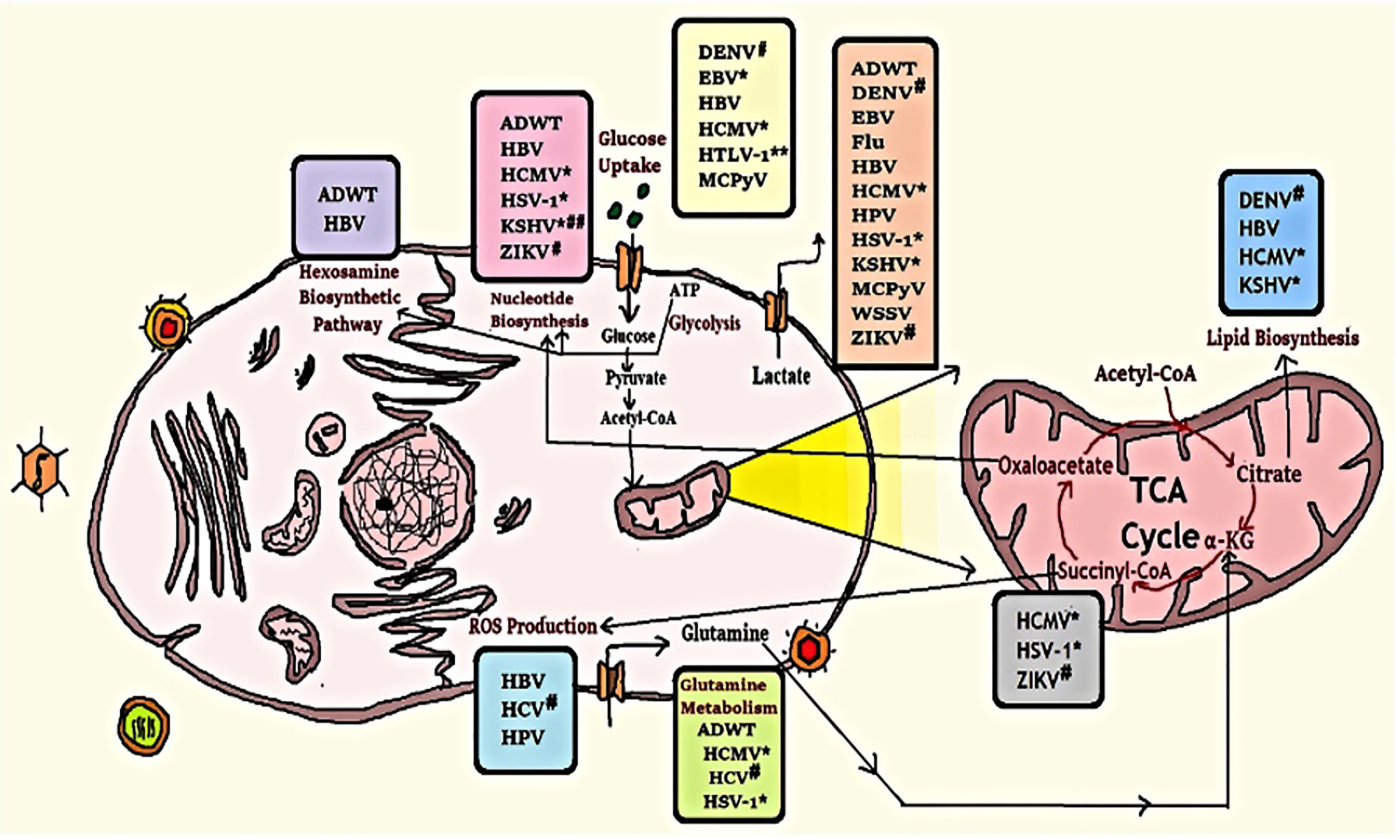
Modification of some metabolic pathways due to viral infection. Human cell metabolism systems alter by viral infection as they rely on host cell machinery to multiply; viruses promote human anabolism for the generation of macromolecules for replication and assembly. *Herpes-virus family; #Flavivirus family; **down-regulates this metabolic activity; ##up-regulates lipid synthesis but down-regulates cholesterol synthesis (199).

Metabolites are those small molecules that are intermediate or end products of metabolism. They have a wide variety of functions, including- signalling, stimulatory and inhibitory effects on enzymes, modulating the immune response, catalytic activity of their own, defense, and interactions with other organisms. Metabolites are used to regulate the growth, differentiation, and activity of the immune system (197). As of now, it is known that COVID-19 impairs those human subjects whose immune system is not efficient enough to fight viral infection. In that case, metabolites can enhance the immune system to counteract viral infection. SARS-CoV-2 has also been reported for manipulating the balance and secretion of metabolites in the host to facilitate their replication process (198), the mechanism of which has been depicted in **Figure 4**.

Some anaerobic commensal bacteria in the cecum and colon produces short-chain fatty acids (SCFAs) (e.g., acetate, propionate, butyrate, etc.) where some Bacteroidetes were reported to produce acetate and propionate, butyrate is primarily made by certain **Firmicutes** (200). Administration of SCFAs alters hematopoiesis to increase the myeloid output, which eventually aids in clearing systemic infections and alleviates allergic reactions (201).

Tryptophan, a substrate for microbial metabolism, produces indole-3-aldehyde, a ligand for the aryl hydrogen receptor (AhR) (202). In this way, it enhances immune maturation at the tissuelevel. AhR expression is required for the expansion of RORγt+ group 3 innate lymphoid cells (ILC3s) (203). It is also pivotal for maintaining the epithelial barrier and homeostasis of intraepithelial lymphocytes (IELs) (204).

Long-chain polyunsaturated fatty acids (PUFAs) have important roles in inflammatory and adaptive immune responses. In most situations, omega-3 and omega-6 PUFAs promote anti-inflammatory and proinflammatory action (205). Furthermore, protein D1, a lipid mediator generated from omega-3 PUFA, may dramatically decrease influenza virus proliferation *via* RNA export machinery (206). Food sources for Omega-3 polyunsaturated fatty acids (PUFAs) (114) are- meat, poultry, cereal-based products, milk products, vegetables, savory sauces and condiments, sugar products and dishes, and special dietary foods.

The Thymosin alpha 1 (Tα1) is a peptide that consists of 28 amino acids (thymalfasin, trade name ZADAXIN ^®^) (207). The amino acid series is homologous in bovine, murine, and human species and a chemical-synthesized version demonstrated close behavior to the native peptide in T-cell maturation modulation (208). Tα1 can influence the mobilization of pre-NK cells that then become cytotoxic following interferon exposure. It has an unusual capacity to maintain the homeostasis of the immune system. Tα1 can also be used as an immune stimulator in SARS patients, and it has successfully managed disease spread (209).

Thymopentin is a pentapeptide generic metabolite having the active site of the naturally found thymopoietin hormone and has immune amplifying effects. It increases thymic T cell development and may help regain immunocompetence in immunosuppressed subjects (210). Myristic acid-modified thymopentin for improved plasma stability and immunomodulation (211). Thymopentin may also be a good alternative for handling this novel coronavirus by improving the immune system.

Levamisole is a synthetic low-molecular-weight organic compound, that can improve immunity. However, levamisole may function either as an immune stimulant or an immunosuppressant depending on the dose and timing. Levamisole and *in vitro* ascorbic acid therapy may restore the lymphocyte subpopulation of the distressed helper/inducer in measles (212). Therefore, the use of levamisole could also be considered for COVID-19 treatment.

Cyclosporine is a cyclic peptide with an immunosuppressive preferential action. Cyclosporine A is used to cure inflammatory diseases. It has played a significant role in viral infection, either promoting or inhibiting their replication (213). Additionally, cyclosporine A prevents replication of all genera of coronavirus, including SARS-CoVs and avian infectious bronchitis virus (214). Therefore, the non-immunosuppressive variants of cyclosporine A may act as broad-range coronavirus inhibitors that are relevant against the new novel virus such as SARS-CoV-2.

## Personal Habits as a Key Tool to Rejuvenate the Immune System

**Figure 5:**
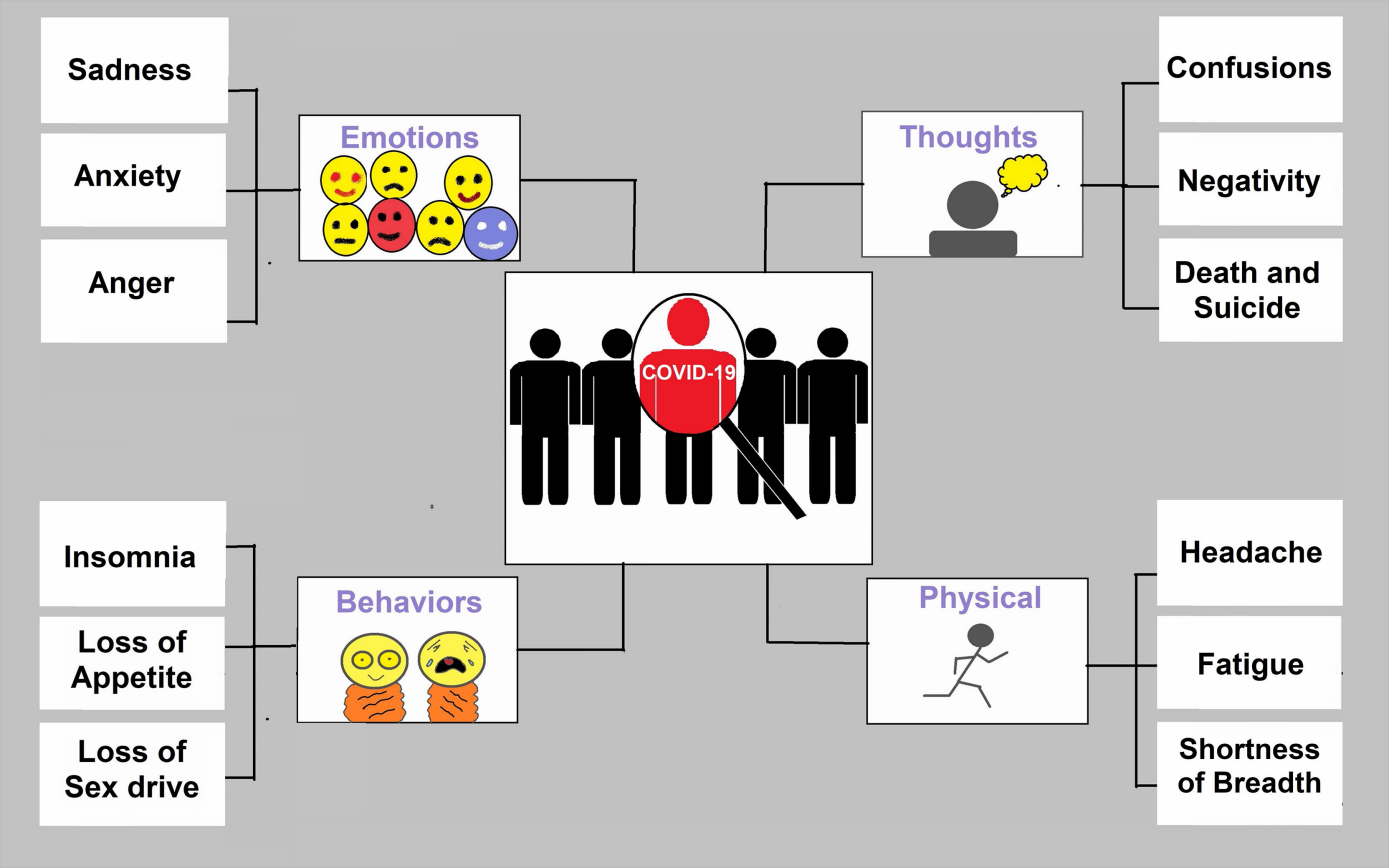
Symptoms of depression for COVID-19 patients. COVID-19 has changed emotions, behaviours, thoughts, and physical condition. Anxiety and stress are the common outcomes in pandemics.

**Exercise or physical activity** is like a safe living medication for chronic illness prevention, recovery, and also proper maintenance of physical and mental condition (52). Routine exercise has demonstrated an improved response to antioxidants that can increase immunosurveillance (**Figure 5**) (215). IL-6, which has an anti-inflammatory function, also partly mediates the immunomodulating effect of exercise (216). The metabolic activity is also associated with daily workouts, including developments in glucose metabolism, lipids, and insulins, and as an adjuvant to immune and metabolic help (217). Physical activity has also been found to alleviate many viral infections, like- influenza, rhinovirus, and herpes viruses like herpes simplex virus 1 (HSV-1), varicella-zoster (VZV), and Epstein-Barr virus (EBV) (218, 219). Moreover, World Health Organization (WHO) has already proposed a guideline that suggests a certain amount of exercises such as walking, breathing for the people who have no symptoms or diagnosis of acute respiratory illness during self-quarantine (220).

Sleep, according to several studies, sleep plays a vital role in the uplift of the immune system (42, 53). Furthermore, when people do not get enough sleep, their antibodies and defensive cells are decreased (221). Sleeping time is closely associated with gender, age, and physical activity, with the ideal, suggested sleep length in adults often ranging between 7 and 9 hours each night (222). In a recent study, 164 individuals took a dose of the common cold and then followed up on their sleeping patterns. The findings revealed that those who have slept for less than six hours a night are more susceptible than those who have slept for more than seven hours to establish cold symptoms, and like so, having enough sleep could help us against the COVID-19 (223). The study indicates that cold and influenza-infected individuals experience worse symptomatic conditions when they sleep badly. This happens may be due to high proinflammatory cytokines, which intervene with T-cells as well as other immune cells (221).

Intermittent Fasting (IF) has been found to affect immunity through changes in various associated components, such as oxidative stress and inflammation, metabolism, weight gain, and feed structure. This food restriction technique can be implemented directly (by the activation of the immune response) (54) or indirectly (through autophagy induction) (224, 225) or by stimulating body monitoring systems and enhancing immunity to cope with stress thus promoting host protection. IF aids in the normalization of the body’s systemic inflammatory status by restricting proinflammatory cytokines (IL-1, IL-6, and TNF-α) (226). Fasting also up regulates gene expression of cytokines type 2 (Il-4, Il-5, and Il-13), crucial for M2 macrophage polarisation (anti-inflammatory) (227). Furthermore, IF can also modulate the potential SARS-CoV-2 immune evading mechanism, which involves ORF3a viral-mediated persistent activation of the NLRP3. Despite being supported by several experimental test results; robust studies are still in need to prove the potential of regulated fasting for the improvement of immunity.

Alkaline diet, the diet or culinary element with a low net potential renal acid load (PRAL) is regarded as an alkaline diet that causes mild alkalosis in the blood, which denatures the virus and helps to control the infection (228). Fruits (raisins, oranges, strawberries, watermelons), juices (apple, lemon, grape) vegetables (spinach, carrot, cabbage, celery, cauliflower, cucumber, radish, green capsicum, tomatoes) milk products (curd, buttermilk) all have a very low PRAL value and have been reported to be effective anti-influenza agents. Current evidence indicates that an alkaline diet can help improve resistance and may even play a role in COVID-19 treatments (229). It raises cellular pH (especially in respiratory cells), preventing viral nucleic acid from reaching the cytoplasm and hence preventing growth and spread (196). Alkaline diets or drinks cause decreased metabolic alkalosis in the cytoplasmic pH of respiratory tract cells, which eventually raises the pH of the endosome, disrupting the viral life cycle in the early phases and partially assisting in the development of physiological resistance to viral infection (229). Nitric Oxide (NO) inhibits viral protein and RNA, as well as NO synthase reduces progeny virus output by 82%, reducing corona virus replication by virtue of its antiviral impact. It is hypothesized that, in addition to humming, eating alkaline meals high in dietary nitrates, which the body can convert to NO, boosts NO expression and carbon dioxide through longer expiration, preventing coagulopathies and morbidity caused by COVID-19 (230). Fresh juice free of preservatives from the aforementioned vegetables, or antioxidants derived from purple carrot and cabbage, in combination with appropriate anti-coagulants, may help prevent or mitigate the negative effects of COVID-19 pathological outcomes, as well as mitigate multi-organ damage caused by COVID-19 during the ongoing pandemic (231).

## Mental Health and Immunity

Stress, worry, depressive symptoms (**Figure 5**), sleeplessness, denial, rage, and terror are among the mental health concerns linked to the COVID-19 pandemic (232) where in some cases COVID-19 may lead to suicide (233–235). During the COVID-19 epidemic, a group of people relies on electronic devices and social media, which upsurges the risk of stress, worry, and anxiety (113, 139). Children and young people, in particular, have limited access to open spaces and playgrounds, owing to their increased reliance on electronic devices such as cell phones. These circumstances have the potential to result in serious mental disorders in the future (236, 237). Researchers should start psychological examinations to detect mental health issues to give early treatment and psychological help. To improve mental health condition, WHO recommended some guidelines to eat a healthy and nutritious diet, limit alcohol consumption, avoid sugary drinks, avoid smoking, 30 minutes physical exercise, listen to music, read the book or play a game (https://www.who.int/).

## Conclusion

An immunostimulant host, rather than a susceptible one, can assure a robust immune system that can fight against COVID-19 infection as well as other viruses or bacteria, by eating a nutritious diet, sleeping well, and exercising often. People who eat a well-balanced diet appear to have a stronger immune system and a lower risk during COVID-19 infection. COVID-19, like many other viral diseases, poses a plethora of challenges to deal with using conventional anti-viral drugs or newly developed vaccines. Hence, a healthy and active immune system is the sole most important factor that can truly help us in the battle of COVID-19 during this global pandemic. Factors like the inclusion of vege`s and fruits in daily diet, medicinal plants and herbs, vitamins, metabolites, probiotics, potential antiviral drugs, and physical exercise to gain a more protective immune response against SARS-CoV-2 have been briefly discussed throughout this review. These elements of boosting immunity can be easily implemented into the daily lifestyle & regular habits that will ultimately ensure a healthy immune system. Besides, It is necessary to move forward with research into the development of potential COVID-19 drugs and vaccines.

This review clearly demonstrates that adequate nutraceuticals and phytochemicals derived from a variety of plant or herb sources can boost up against COVID-19 infection. Therefore, a lack of immunity intensifiers can be detrimental to the immune system. To summarize, the simplest approach to strengthen the immune system is to consume nutrients that act as immunity boosters, and phytochemicals are the best after nutrients, and both can be utilized as a potent weapon against SARS-CoV-2.

## Author Contributions

MI and MAH: Writing original draft, review, and editing. MAR, FH, MR and AB: Literature review and editing. AM, TD and SB: Construct figures, tables and data curation. CH and FA: Conceptualization, review, and editing. PB and MJ: Preparing response to the reviewers and involve resource management. All authors contributed to the article and approved the submitted version.

## Funding

This study was funded through a seed grant from the Life Sciences Platform and KTH Water Center, KTH Royal Institute of Technology, Stockholm, Sweden and institutional grants from North South University, Dhaka, Bangladesh and Noakhali Science and Technology University, Noakhali, Bangladesh.

## Conflict of Interest

The authors declare that the research was conducted in the absence of any commercial or financial relationships that could be construed as a potential conflict of interest.

## Publisher’s Note

All claims expressed in this article are solely those of the authors and do not necessarily represent those of their affiliated organizations, or those of the publisher, the editors and the reviewers. Any product that may be evaluated in this article, or claim that may be made by its manufacturer, is not guaranteed or endorsed by the publisher.

## References

[1] Kahn JS McIntosh K. History and Recent Advances in Coronavirus Discovery. Pediatr Infect Dis J (2005) 24:223–7. doi: 10.1097/01.inf.0000188166.17324.60 16378050

[2] Mahase E. Covid-19: Coronavirus was First Described in The BMJ in 1965. BMJ (2020) 369:m1547. doi: 10.1136/bmj.m1547 32299810

[3] Woodhead M Ewig S Torres A. Severe Acute Respiratory Syndrome (SARS). Eur Respir J (2003) 21:739–40. doi: 10.1183/09031936.03.00035403 12765412

[4] Remembering SARS: A Deadly Puzzle and the Efforts to Solve It. Centers for Disease Control and Prevention. CDC. 26 Apr. 2013. Available at: http://www.cdc.gov/about/history/sars/feature.htm

[5] WHO. Coronavirus Never Before Seen in Humans is the Cause of SARS N.D. World Health Organization (2003). Available at: https://www.who.int/news/item/16-04-2003-update-31---coronavirus-never-before-seen-in-humans-is-the-cause-of-sars.

[6] van der Hoek L Pyrc K Jebbink MF Vermeulen-Oost W Berkhout RJM Wolthers KC. Identification of a New Human Coronavirus. Nat Med (2004) 10:368–73. doi: 10.1038/nm1024 PMC7095789 15034574

[7] Zaki AM van Boheemen S Bestebroer TM Osterhaus ADME Fouchier RAM. Isolation of a Novel Coronavirus From a Man With Pneumonia in Saudi Arabia. N Engl J Med (2012) 367:1814–20. doi: 10.1056/NEJMoa1211721 23075143

[8] Wang C Horby PW Hayden FG Gao GF. A Novel Coronavirus Outbreak of Global Health Concern. Lancet (2020) 395:470–3. doi: 10.1016/S0140-6736(20)30185-9 PMC7135038 31986257

[9] Jiang F Deng L Zhang L Cai Y Cheung CW Xia Z. Review of the Clinical Characteristics of Coronavirus Disease 2019 (COVID-19). J Gen Intern Med (2020) 35:1545–9. doi: 10.1007/s11606-020-05762-w PMC7088708 32133578

[10] Fontanet A Autran B Lina B Kieny MP Karim SSA Sridhar D. SARS-CoV-2 Variants and Ending the COVID-19 Pandemic. Lancet (2021) 397:952–4. doi: 10.1016/S0140-6736(21)00370-6 PMC7906631 33581803

[11] Liu Y Liu J Plante KS Plante JA Xie X Zhang X. The N501Y Spike Substitution Enhances SARS-CoV-2 Transmission. bioRxiv Preprint (2021) 602:294–9. doi: 10.1101/2021.03.08.434499 PMC8900207 34818667

[12] Wong NA Saier MH. The SARS-Coronavirus Infection Cycle: A Survey of Viral Membrane Proteins, Their Functional Interactions and Pathogenesis. Int J Mol Sci (2021) 22:1308. doi: 10.3390/ijms22031308 33525632 PMC7865831

[13] V’kovski P Kratzel A Steiner S Stalder H Thiel V. Coronavirus Biology and Replication: Implications for SARS-CoV-2. Nat Rev Microbiol (2021) 19:155–70. doi: 10.1038/s41579-020-00468-6 PMC7592455 33116300

[14] Parasher A. COVID-19: Current Understanding of its Pathophysiology, Clinical Presentation and Treatment. Postgrad Med J (2021) 97:312–20. doi: 10.1136/postgradmedj-2020-138577 PMC10017004 32978337

[15] Trougakos IP Stamatelopoulos K Terpos E Tsitsilonis OE Aivalioti E Paraskevis D. Insights to SARS-CoV-2 Life Cycle, Pathophysiology, and Rationalized Treatments That Target COVID-19 Clinical Complications. J BioMed Sci (2021) 28:9. doi: 10.1186/s12929-020-00703-5 33435929 PMC7801873

[16] Shereen MA Khan S Kazmi A Bashir N Siddique R. COVID-19 Infection: Origin, Transmission, and Characteristics of Human Coronaviruses. J Adv Res (2020) 24:91–8. doi: 10.1016/j.jare.2020.03.005 PMC7113610 32257431

[17] Yashavantha RHC Chelliah J. The Emergence of a Novel Coronavirus (SARS-CoV-2) Disease and Their Neuroinvasive Propensity may Affect in COVID-19 Patients. J Med Virol (2020) 92:786–90. doi: 10.1002/jmv.25918 PMC7264535 32320066

[18] Yin W Mao C Luan X Shen D-D Shen Q Su H. Structural Basis for Inhibition of the RNA-Dependent RNA Polymerase From SARS-CoV-2 by Remdesivir. Science (2020) 368:1499–504. doi: 10.1126/science.abc1560 PMC7199908 32358203

[19] Pokhrel R Chapagain P Siltberg-Liberles J. Potential RNA-Dependent RNA Polymerase Inhibitors as Prospective Therapeutics Against SARS-CoV-2. J Med Microbiol (2020) 69:864–73. doi: 10.1099/jmm.0.001203 PMC7451031 32469301

[20] Adhikari P Li N Shin M Steinmetz NF Twarock R Podgornik R. Intra- and Intermolecular Atomic-Scale Interactions in the Receptor Binding Domain of SARS-CoV-2 Spike Protein: Implication for ACE2 Receptor Binding. Phys Chem Chem Phys (2020) 22:18272–83. doi: 10.1039/D0CP03145C 32756685

[21] Schoeman D Fielding BC. Coronavirus Envelope Protein: Current Knowledge. Virol J (2019) 16:69. doi: 10.1186/s12985-019-1182-0 31133031 PMC6537279

[22] Singh E Khan RJ Jha RK Amera GM Jain M Singh RP. A Comprehensive Review on Promising Anti-Viral Therapeutic Candidates Identified Against Main Protease From SARS-CoV-2 Through Various Computational Methods. J Genet Eng Biotechnol (2020) 18:69. doi: 10.1186/s43141-020-00085-z 33141358 PMC7607901

[23] Goyal B Goyal D. Targeting the Dimerization of the Main Protease of Coronaviruses: A Potential Broad-Spectrum Therapeutic Strategy. ACS Comb Sci (2020) 22:297–305. doi: 10.1021/acscombsci.0c00058 32402186

[24] Huang C Wang Y Li X Ren L Hu Y Hu Y. Clinical Features of Patients Infected With 2019 Novel Coronavirus in Wuhan, China. Lancet (2020) 395:497–506. doi: 10.1016/S0140-6736(20)30183-5 PMC7159299 31986264

[25] Lai C-C Shih T-P Ko W-C Tang H-J Hsueh P-R. Severe Acute Respiratory Syndrome Coronavirus 2 (SARS-CoV-2) and Coronavirus Disease-2019 (COVID-19): The Epidemic and the Challenges. Int J Antimicrob Agents (2020) 55:105924. doi: 10.1016/j.ijantimicag.2020.105924 32081636 PMC7127800

[26] Chen N Zhou M Dong X Qu J Gong F Han Y. Epidemiological and Clinical Characteristics of 99 Cases of 2019 Novel Coronavirus Pneumonia in Wuhan, China: A Descriptive Study. Lancet (2020) 395:507–13. doi: 10.1016/S0140-6736(20)30211-7 PMC7135076 32007143

[27] Guan W Ni Z Hu Y Liang W Ou C He J. Clinical Characteristics of Coronavirus Disease 2019 in China. N Engl J Med (2020) 382:1708–20. doi: 10.1056/NEJMoa2002032 PMC7092819 32109013

[28] Forni G Mantovani A. COVID-19 Vaccines: Where We Stand and Challenges Ahead. Cell Death Differ (2021) 28:626–39. doi: 10.1038/s41418-020-00720-9 PMC7818063 33479399

[29] Forni G Mantovani A Moretta L Rezza G. Vaccines. Accademia Nazionale dei Lincei (2018). Available at: https://www.lincei.it/it/article/i-vaccini-vaccines-position-paper.

[30] Cevik M Tate M Lloyd O Maraolo AE Schafers J Ho A. SARS-CoV-2, SARS-CoV, and MERS-CoV Viral Load Dynamics, Duration of Viral Shedding, and Infectiousness: A Systematic Review and Meta-Analysis. Lancet Microbe (2021) 2:e13–22. doi: 10.1016/S2666-5247(20)30172-5 PMC7837230 33521734

[31] Rahman MA Islam MS. Early Approval of COVID-19 Vaccines: Pros and Cons. Hum Vaccin Immunother (2021) 17:3288–96. doi: 10.1080/21645515.2021.1944742 PMC8437465 34283001

[32] Pathan RK Biswas M Khandaker MU. Time Series Prediction of COVID-19 by Mutation Rate Analysis Using Recurrent Neural Network-Based LSTM Model. Chaos Solitons Fractals (2020) 138:110018. doi: 10.1016/j.chaos.2020.110018 32565626 PMC7293453

[33] Chen J. Should the World Collaborate Imminently to Develop Neglected Live-Attenuated Vaccines for COVID-19? J Med Virol (2022) 94:82–7. doi: 10.1002/jmv.27335 PMC8662152 34524688

[34] Rosen B Waitzberg R Israeli A Hartal M Davidovitch N. Addressing Vaccine Hesitancy and Access Barriers to Achieve Persistent Progress in Israel’s COVID-19 Vaccination Program. Isr J Health Policy Res (2021) 10:43. doi: 10.1186/s13584-021-00481-x 34340714 PMC8326649

[35] Gilbert PB Montefiori DC McDermott AB Fong Y Benkeser D Deng W. Immune Correlates Analysis of the mRNA-1273 COVID-19 Vaccine Efficacy Clinical Trial. Science (2022) 375:43–50. doi: 10.1126/science.abm3425 34812653 PMC9017870

[36] Wang J Pan L Tang S Ji JS Shi X. Mask Use During COVID-19: A Risk Adjusted Strategy. Environ Pollut (2020) 266:115099. doi: 10.1016/j.envpol.2020.115099 32623270 PMC7314683

[37] Lee C Chen Y Wu P Chiou W. An Unintended Consequence of Social Distance Regulations: COVID-19 Social Distancing Promotes the Desire for Money. Br J Psychol (2021) 112:866–78. doi: 10.1111/bjop.12497 PMC8013677 33615446

[38] Khan MH Yadav H. Sanitization During and After COVID-19 Pandemic: A Short Review. Trans Indian Natl Acad Eng (2020) 5:617–27. doi: 10.1007/s41403-020-00177-9

[39] Arenas MD Villar J González C Cao H Collado S Crespo M. Management of the SARS-CoV-2 (COVID-19) Coronavirus Epidemic in Hemodialysis Units. Nefrol (2020) 40:258–64. doi: 10.1016/j.nefroe.2020.04.001 PMC7142670 32340751

[40] Gershoff SN Vitamin C. (Ascorbic Acid): New Roles, New Requirements? Nutr Rev (2009) 51:313–26. doi: 10.1111/j.1753-4887.1993.tb03757.x 8108031

[41] Wahlstrom KL Berger AT Widome R. Relationships Between School Start Time, Sleep Duration, and Adolescent Behaviors. Sleep Heal (2017) 3:216–21. doi: 10.1016/j.sleh.2017.03.002 PMC7178613 28526260

[42] Besedovsky L Lange T Born J. Sleep and Immune Function. Pflügers Arch - Eur J Physiol (2012) 463:121–37. doi: 10.1007/s00424-011-1044-0 PMC3256323 22071480

[43] Nizami NS Uddin CSM. Strong Immunity- A Major Weapon to Fight Against Covid-19. IOSR J Pharm Biol Sci (2020) 15:22–29. doi: 10.9790/3008-1503032229

[44] Calder PC. Nutrition, Immunity and COVID-19. BMJ Nutr Prev Heal (2020) 3:74–92. doi: 10.1136/bmjnph-2020-000085 PMC7295866 33230497

[45] Bhaskaram P. Immunobiology of Mild Micronutrient Deficiencies. Br J Nutr (2001) 85:S75–80. doi: 10.1079/bjn2000297 11509093

[46] Wintergerst ES Maggini S Hornig DH. Contribution of Selected Vitamins and Trace Elements to Immune Function. Ann Nutr Metab (2007) 51:301–23. doi: 10.1159/000107673 17726308

[47] Li G Fan Y Lai Y Han T Li Z Zhou P. Coronavirus Infections and Immune Responses. J Med Virol (2020) 92:424–32. doi: 10.1002/jmv.25685 PMC7166547 31981224

[48] de Wilde AH Snijder EJ Kikkert M van Hemert MJ. Host Factors in Coronavirus Replication. Curr Top Microbiol Immunol (2018) 419:1–42. doi: 10.1007/82_2017_25 28643204 PMC7119980

[49] Deeks SG Odorizzi PM Sekaly RP. The Interferon Paradox: Can Inhibiting an Antiviral Mechanism Advance an HIV Cure? J Clin Invest (2017) 127:103–5. doi: 10.1172/JCI91916 PMC5199692 27941242

[50] Channappanavar R Zhao J Perlman S. T Cell-Mediated Immune Response to Respiratory Coronaviruses. Immunol Res (2014) 59:118–28. doi: 10.1007/s12026-014-8534-z PMC4125530 24845462

[51] Chen G Wu D Guo W Cao Y Huang D Wang H. Clinical and Immunological Features of Severe and Moderate Coronavirus Disease 2019. J Clin Invest (2020) 130:2620–9. doi: 10.1172/JCI137244 PMC7190990 32217835

[52] Fletcher GF Landolfo C Niebauer J Ozemek C Arena R Lavie CJ. Promoting Physical Activity and Exercise: JACC Health Promotion Series. J Am Coll Cardiol (2018) 72:1622–39. doi: 10.1016/j.jacc.2018.08.2141 30261965

[53] Lange T Dimitrov S Born J. Effects of Sleep and Circadian Rhythm on the Human Immune System. Ann N Y Acad Sci (2010) 1193:48–59. doi: 10.1111/j.1749-6632.2009.05300.x 20398008

[54] Cheng C-W Adams GB Perin L Wei M Zhou X Lam BS. Prolonged Fasting Reduces IGF-1/PKA to Promote Hematopoietic-Stem-Cell-Based Regeneration and Reverse Immunosuppression. Cell Stem Cell (2014) 14:810–23. doi: 10.1016/j.stem.2014.04.014 PMC4102383 24905167

[55] Institute of Medicine. Dietary Reference Intakes for Energy, Carbohydrate, Fiber, Fat, Fatty Acids, Cholesterol, Protein, and Amino Acids. Washington, D.C: National Academies Press (2005). doi: 10.17226/10490

[56] Institute of Medicine. Dietary Reference Intakes for Water, Potassium, Sodium, Chloride, and Sulfate. Washington, D.C: National Academies Press (2005). doi: 10.17226/10925

[57] EFSA Panel on Dietetic Products, Nutrition, and Allergies (NDA). Scientific Opinion on Dietary Reference Values for Water. EFSA J (2010) 8:1–48. doi: 10.2903/j.efsa.2010.1459

[58] Huang Z Liu Y Qi G Brand D Zheng S. Role of Vitamin A in the Immune System. J Clin Med (2018) 7:258. doi: 10.3390/jcm7090258 PMC6162863 30200565

[59] Shankar AH Prasad AS. Zinc and Immune Function: The Biological Basis of Altered Resistance to Infection. Am Soc Nutr (1998) 68:447S–63S. doi: 10.1093/ajcn/68.2.447S 9701160

[60] Fraker PJ King LE Laakko T Vollmer TL. The Dynamic Link Between the Integrity of the Immune System and Zinc Status. J Nutr (2000) 130:1399S–406S. doi: 10.1093/jn/130.5.1399S 10801951

[61] Zeng HL Yang Q Yuan P Wang X Cheng L. Associations of Essential and Toxic Metals/Metalloids in Whole Blood With Both Disease Severity and Mortality in Patients With COVID-19. FASEB J (2021) 35:1–12. doi: 10.1096/fj.202002346RR PMC7995111 33577131

[62] Bayan L Koulivand PH Gorji A. Garlic: A Review of Potential Therapeutic Effects. Avicenna J Phytomedicine (2014) 4:1–14. doi: 10.22038/ajp.2014.1741 PMC4103721 25050296

[63] Arreola R Quintero-Fabián S López-Roa RI Flores-Gutiérrez EO Reyes-Grajeda JP Carrera-Quintanar L. Immunomodulation and Anti-Inflammatory Effects of Garlic Compounds. J Immunol Res (2015) 2015:1–13. doi: 10.1155/2015/401630 PMC4417560 25961060

[64] Donma MM Donma O. The Effects of Allium Sativum on Immunity Within the Scope of COVID-19 Infection. Med Hypotheses (2020) 144:1–5. doi: 10.1016/J.MEHY.2020.109934 PMC7265825 32512493

[65] Rasool A Khan M-U-R Ali MA Anjum AA Ahmed I Aslam A. Anti-Avian Influenza Virus H9N2 Activity of Aqueous Extracts of Zingiber Officinalis (Ginger) and Allium Sativum (Garlic) in Chick Embryos. Pak J Pharm Sci (2017) 30:1341–4. 29039335

[66] Chang JS Wang KC Yeh CF Shieh DE Chiang LC. Fresh Ginger (Zingiber Officinale) has Anti-Viral Activity Against Human Respiratory Syncytial Virus in Human Respiratory Tract Cell Lines. J Ethnopharmacol (2013) 145:146–51. doi: 10.1016/j.jep.2012.10.043 23123794

[67] Arora R Chawla R Marwah R Arora P Sharma RK Kaushik V. Potential of Complementary and Alternative Medicine in Preventive Management of Novel H1N1 Flu (Swine Flu) Pandemic: Thwarting Potential Disasters in the Bud. Evid Based Complement Alternat Med (2011) 2011:1–16. doi: 10.1155/2011/586506 PMC2957173 20976081

[68] Tripathi S Bruch D Kittur DS. Ginger Extract Inhibits LPS Induced Macrophage Activation and Function. BMC Complement Altern Med (2008) 8:1. doi: 10.1186/1472-6882-8-1 18173849 PMC2234390

[69] Pilau MR Alves SH Weiblen R Arenhart S Cueto AP Lovato LT. Antiviral Activity of the Lippia Graveolens (Mexican Oregano) Essential Oil and its Main Compound Carvacrol Against Human and Animal Viruses. Braz J Microbiol (2011) 42:1616–24. doi: 10.1590/S1517-83822011000400049 PMC3768712 24031796

[70] Sharifi-Rad J Salehi B Schnitzler P Ayatollahi SA Kobarfard F Fathi M. Susceptibility of Herpes Simplex Virus Type 1 to Monoterpenes Thymol, Carvacrol, P-Cymene and Essential Oils of Sinapis Arvensis L., Lallemantia Royleana Benth. And Pulicaria Vulgaris Gaertn. Cell Mol Biol (2017) 63:42–7. doi: 10.14715/cmb/2017.63.8.10 28886313

[71] Mondal S Varma S Bamola VD Naik SN Mirdha BR Padhi MM. Double-Blinded Randomized Controlled Trial for Immunomodulatory Effects of Tulsi (Ocimum Sanctum Linn.) Leaf Extract on Healthy Volunteers. J Ethnopharmacol (2011) 136:452–6. doi: 10.1016/j.jep.2011.05.012 21619917

[72] Chiang L-C Ng L-T Cheng P-W Chiang W Lin C-C. Antiviral Activities of Extracts and Selected Pure Constituents of Ocimum Basilicum. Clin Exp Pharmacol Physiol (2005) 32:811–6. doi: 10.1111/j.1440-1681.2005.04270.x 16173941

[73] Hamidpour M Hamidpour R Hamidpour S Shahlari M. Chemistry, Pharmacology, and Medicinal Property of Sage (Salvia) to Prevent and Cure Illnesses Such as Obesity, Diabetes, Depression, Dementia, Lupus, Autism, Heart Disease, and Cancer. J Tradit Complement Med (2014) 4:82–8. doi: 10.4103/2225-4110.130373 PMC4003706 24860730

[74] Ghorbani A Esmaeilizadeh M. Pharmacological Properties of Salvia Officinalis and its Components. J Tradit Complement Med (2017) 7:433–40. doi: 10.1016/j.jtcme.2016.12.014 PMC5634728 29034191

[75] Geuenich S Goffinet C Venzke S Nolkemper S Baumann I Plinkert P. Aqueous Extracts From Peppermint, Sage and Lemon Balm Leaves Display Potent Anti-HIV-1 Activity by Increasing the Virion Density. Retrovirology (2008) 5:1–16. doi: 10.1186/1742-4690-5-27 18355409 PMC2288616

[76] Badgujar SB Patel VV Bandivdekar AH. Foeniculum Vulgare Mill: A Review of its Botany, Phytochemistry, Pharmacology, Contemporary Application, and Toxicology. BioMed Res Int (2014) 2014:1–32. doi: 10.1155/2014/842674 PMC4137549 25162032

[77] Astani A Reichling J Schnitzler P. Screening for Antiviral Activities of Isolated Compounds From Essential Oils. Evidence-Based Complement Altern Med (2011) 2011:1–8. doi: 10.1093/ecam/nep187 PMC3096453 20008902

[78] Lee HS Kang P Kim KY Seol GH. Foeniculum Vulgare Mill. Protects Against Lipopolysaccharide-Induced Acute Lung Injury in Mice Through ERK-Dependent NF-kB Activation. Korean J Physiol Pharmacol (2015) 19:183–9. doi: 10.4196/kjpp.2015.19.2.183 PMC4342739 25729281

[79] Pourghanbari G Nili H Moattari A Mohammadi A Iraji A. Antiviral Activity of the Oseltamivir and Melissa Officinalis L. Essential Oil Against Avian Influenza A Virus (H9N2). Virusdisease (2016) 27:170–8. doi: 10.1007/s13337-016-0321-0 PMC4908999 27366768

[80] Schnitzler P Schuhmacher A Astani A Reichling J. Melissa Officinalis Oil Affects Infectivity of Enveloped Herpesviruses. Phytomedicine (2008) 15:734–40. doi: 10.1016/j.phymed.2008.04.018 18693101

[81] Astani A Navid MH Schnitzler P. Attachment and Penetration of Acyclovir-Resistant Herpes Simplex Virus are Inhibited by Melissa Officinalis Extract. Phytother Res (2014) 28:1547–52. doi: 10.1002/ptr.5166 24817544

[82] Chen S-G Leu Y-L Cheng M-L Ting SC Liu C-C Wang S-D. Anti-Enterovirus 71 Activities of Melissa Officinalis Extract and its Biologically Active Constituent Rosmarinic Acid. Sci Rep (2017) 7:12264. doi: 10.1038/s41598-017-12388-2 28947773 PMC5613005

[83] Rahman MA Ueda K Honda T. A Traditional Chinese Medicine, Maoto, Suppresses Hepatitis B Virus Production. Front Cell Infect Microbiol (2021) 10:581345. doi: 10.3389/fcimb.2020.581345 33553000 PMC7862555

[84] Wang L Yang R Yuan B Liu Y Liu C. The Antiviral and Antimicrobial Activities of Licorice, a Widely-Used Chinese Herb. Acta Pharm Sin B (2015) 5:310–5. doi: 10.1016/j.apsb.2015.05.005 PMC4629407 26579460

[85] Yoshino T Arita R Horiba Y Watanabe K. The Use of Maoto (Ma-Huang-Tang), a Traditional Japanese Kampo Medicine, to Alleviate Flu Symptoms: A Systematic Review and Meta-Analysis. BMC Complement Altern Med (2019) 19:68. doi: 10.1186/s12906-019-2474-z 30885188 PMC6421694

[86] Feng Yeh C Chih Wang K Chai Chiang L Shieh DE Hong Yen M San Chang J. Water Extract of Licorice had Anti-Viral Activity Against Human Respiratory Syncytial Virus in Human Respiratory Tract Cell Lines. J Ethnopharmacol (2013) 148:466–73. doi: 10.1016/j.jep.2013.04.040 PMC7126896 23643542

[87] Fukuchi K Okudaira N Adachi K Odai-Ide R Watanabe S Ohno H. Antiviral and Antitumor Activity of Licorice Root Extracts. In Vivo (Brooklyn) (2016) 30:777–85. doi: 10.21873/invivo.10994 27815461

[88] Cinatl J Morgenstern B Bauer G Chandra P Rabenau H Doerr HW. Glycyrrhizin, an Active Component of Liquorice Roots, and Replication of SARS-Associated Coronavirus. Lancet (2003) 361:2045–6. doi: 10.1016/S0140-6736(03)13615-X PMC7112442 12814717

[89] Jia Y-Y Guan R-F Wu Y-H Yu X-P Lin W-Y Zhang Y-Y. Taraxacum Mongolicum Extract Exhibits a Protective Effect on Hepatocytes and an Antiviral Effect Against Hepatitis B Virus in Animal and Human Cells. Mol Med Rep (2014) 9:1381–7. doi: 10.3892/mmr.2014.1925 24481875

[90] Han H He W Wang W Gao B. Inhibitory Effect of Aqueous Dandelion Extract on HIV-1 Replication and Reverse Transcriptase Activity. BMC Complement Altern Med (2011) 11:1–10. doi: 10.1186/1472-6882-11-112 22078030 PMC3261818

[91] He W Han H Wang W Gao B. Anti-Influenza Virus Effect of Aqueous Extracts From Dandelion. Virol J (2011) 8:1–11. doi: 10.1186/1743-422X-8-538 22168277 PMC3265450

[92] Meireles D Gomes J Lopes L Hinzmann M Machado J. A Review of Properties, Nutritional and Pharmaceutical Applications of Moringa Oleifera: Integrative Approach on Conventional and Traditional Asian Medicine. Adv Tradit Med (2020) 20:495–515. doi: 10.1007/s13596-020-00468-0

[93] Gasmalbari E Elobeid A Abbadi O. The Use of Traditional Medicines, Vitamins, and Minerals Against COVID - 19. Int J Recent Res Life Sci (2020) 7:15–24. Available at: https://www.paperpublications.org/issue/IJRRLS/Issue-4-October-2020-December-2020.

[94] Sachdeva V Roy A Bharadvaja N. Current Prospects of Nutraceuticals: A Review. Curr Pharm Biotechnol (2020) 21:884–96. doi: 10.2174/1389201021666200130113441 32000642

[95] Kulyar MF-A Li R Mehmood K Waqas M Li K Li J. Potential Influence of Nagella Sativa (Black Cumin) in Reinforcing Immune System: A Hope to Decelerate the COVID-19 Pandemic. Phytomedicine (2020) 85:1–8. doi: 10.1016/j.phymed.2020.153277 PMC7347483 32773257

[96] Oladele JO Ajayi EI Oyeleke OM Oladele OT Olowookere BD Adeniyi BM. A Systematic Review on COVID-19 Pandemic With Special Emphasis on Curative Potentials of Nigeria Based Medicinal Plants. Heliyon (2020) 6:1–17. doi: 10.1016/j.heliyon.2020.e04897 PMC7480258 32929412

[97] Vellingiri B Jayaramayya K Iyer M Narayanasamy A Govindasamy V Giridharan B. COVID-19: A Promising Cure for the Global Panic. Sci Total Environ (2020) 725:1–18. doi: 10.1016/j.scitotenv.2020.138277 PMC7128376 32278175

[98] Khanna K Kohli SK Kaur R Bhardwaj A Bhardwaj V Ohri P. Herbal Immune-Boosters: Substantial Warriors of Pandemic Covid-19 Battle. Phytomedicine (2021) 85:153361. doi: 10.1016/j.phymed.2020.153361 33485605 PMC7532351

[99] Panyod S Ho C-T Sheen L-Y. Dietary Therapy and Herbal Medicine for COVID-19 Prevention: A Review and Perspective. J Tradit Complement Med (2020) 10:420–7. doi: 10.1016/j.jtcme.2020.05.004 PMC7260602 32691006

[100] Liang C Hui N Liu Y Qiao G Li J Tian L. Insights Into Forsythia Honeysuckle (Lianhuaqingwen) Capsules: A Chinese Herbal Medicine Repurposed for COVID-19 Pandemic. Phytomed Plus (2021) 1:100027. doi: 10.1016/j.phyplu.2021.100027 PMC7833308 35399819

[101] Song Z Xu Y Bao L Zhang L Yu P Qu Y. From SARS to MERS, Thrusting Coronaviruses Into the Spotlight. Viruses (2019) 11:59. doi: 10.3390/v11010059 PMC6357155 30646565

[102] Wang B Kovalchuk A Li D Ilnytskyy Y Kovalchuk I Kovalchuk O. In Search of Preventative Strategies: Novel Anti-Inflammatory High-CBD Cannabis Sativa Extracts Modulate ACE2 Expression in COVID-19 Gateway Tissues. Aging (Albany NY) (2020) 12:22425–44. doi: 10.18632/aging.202225 PMC7746344 33221759

[103] Chowdhury MA Shahid MA Kashem MA. Scope of Natural Plant Extract to Deactivate COVID-19. Eur PM (2020). doi: 10.21203/rs.3.rs-19240/v1

[104] Meneguzzo F Ciriminna R Zabini F Pagliaro M. Review of Evidence Available on Hesperidin-Rich Products as Potential Tools Against COVID-19 and Hydrodynamic Cavitation-Based Extraction as a Method of Increasing Their Production. Processes (2020) 8:549. doi: 10.3390/pr8050549

[105] Nagle V Gaikwad M Pawar Y Dasgupta S. Marine Red Alga Porphyridium Sp. As a Source of Sulfated Polysaccharides (SPs) for Combating Against COVID-19. Preprints (2020) 2020040168.

[106] Walter TM Justinraj CS Nandini VS. Effect of Nilavembu Kudineer in the Prevention and Management of COVID–19 by Inhibiting ACE2 Receptor. Siddha Papers (2020) 15(2):531–5.

[107] Salim B Noureddine M. Identification of Compounds From Nigella Sativa as New Potential Inhibitors of 2019 Novel Coronasvirus (Covid-19): Molecular Docking Study. ChemRxiv (2020), 2020040079. doi: 10.20944/preprints202004.0079.v1

[108] Bhatia S Giri S Lal AF Singh S. Battle Against Coronavirus: Repurposing Old Friends (Food Borne Polyphenols) for New Enemy (COVID-19). ChemRxiv (2020). doi: 10.26434/chemrxiv.12108546

[109] Rathinavel T Palanisamy M Palanisamy S Subramanian A Thangaswamy S. Phytochemical 6-Gingerol – A Promising Drug of Choice for COVID-19. Int J Adv Sci Eng (2020) 06:1482–9. doi: 10.29294/ijase.6.4.2020.1482-1489

[110] Adem S Eyupoglu V Sarfraz I Rasul A Ali M. Identification of Potent COVID-19 Main Protease (Mpro) Inhibitors From Natural Polyphenols: An in Silico Strategy Unveils a Hope Against CORONA. Preprints (2020). doi: 10.20944/preprints202003.0333.v1 PMC8957318 35364308

[111] Thuy BTP My TTA Hai NTT Hieu LT Hoa TT Thi Phuong Loan H. Investigation Into SARS-CoV-2 Resistance of Compounds in Garlic Essential Oil. ACS Omega (2020) 5:8312–20. doi: 10.1021/acsomega.0c00772 PMC7123907 32363255

[112] Shaghaghi N. Molecular Docking Study of Novel COVID-19 Protease With Low Risk Terpenoides Compounds of Plants. ChemRxiv (2020). doi: 10.26434/chemrxiv.11935722

[113] Khan S Siddique R Li H Ali A Shereen MA Bashir N. Impact of Coronavirus Outbreak on Psychological Health. J Glob Health (2020) 10:1–6. doi: 10.7189/JOGH.10.010331 PMC7180007 32355556

[114] Sharma AD Kaur I. Jensenone From Eucalyptus Essential Oil as a Potential Inhibitor of COVID 19 Corona Virus Infection. Res Rev Biotechnol Biosci (2020) 7:59–66. doi: 10.5281/zenodo.3748477

[115] Khairan K Idroes R Tallei TE Nasim MJ Jacob C. Bioactive Compounds From Medicinal Plants and Their Possible Effect as Therapeutic Agents Against COVID-19: A Review. Curr Nutr Food Sci (2021) 17:621–33. doi: 10.2174/1573401317999210112201439

[116] Shah K Kamrai D Mekala H Mann B Desai K Patel RS. Focus on Mental Health During the Coronavirus (COVID-19) Pandemic: Applying Learnings From the Past Outbreaks. Cureus (2020) 12:1–6. doi: 10.7759/cureus.7405 PMC7182052 32337131

[117] Jovic TH Ali SR Ibrahim N Jessop ZM Tarassoli SP Dobbs TD. Could Vitamins Help in the Fight Against Covid-19? Nutrients (2020) 12:1–30. doi: 10.3390/nu12092550 PMC7551685 32842513

[118] National Research Council. Recommended Dietary Allowances. Washington, D.C: National Academies Press (1989). doi: 10.17226/1349

[119] Toledano JM Moreno-Fernandez J Puche-Juarez M Ochoa JJ Diaz-Castro J. Implications of Vitamins in COVID-19 Prevention and Treatment Through Immunomodulatory and Anti-Oxidative Mechanisms. Antioxidants (2021) 11:5. doi: 10.3390/antiox11010005 35052509 PMC8773198

[120] Tepasse P-R Vollenberg R Fobker M Kabar I Schmidt H Meier JA. Vitamin A Plasma Levels in COVID-19 Patients: A Prospective Multicenter Study and Hypothesis. Nutrients (2021) 13:2173. doi: 10.3390/nu13072173 34202697 PMC8308355

[121] Sharma L. Dietary Management to Build Adaptive Immunity Against COVID19. J PeerSci (2020) 2:1–6. doi: 10.9790/3008-1503032229

[122] Carr AC Maggini S. Vitamin C and Immune Function. Nutrients (2017) 9:1–25. doi: 10.3390/nu9111211 PMC5707683 29099763

[123] Khan MF Khan MA Khan ZA Ahamad T Ansari WA. Identification of Dietary Molecules as Therapeutic Agents to Combat COVID-19 Using Molecular Docking Studies. preprints (2020). doi: 10.21203/rs.3.rs-19560/v1

[124] Hemilä H de Man AME. Vitamin C and COVID-19. Front Med (2021) 7:559811. doi: 10.3389/fmed.2020.559811 PMC7848027 33537320

[125] Name JJ Souza ACR Vasconcelos AR Prado PS Pereira CPM. Zinc, Vitamin D and Vitamin C: Perspectives for COVID-19 With a Focus on Physical Tissue Barrier Integrity. Front Nutr (2020) 7:606398. doi: 10.3389/fnut.2020.606398 33365326 PMC7750357

[126] Uddin MS Millat MS Baral PK Ferdous M Uddin MG Sarwar MS. The Protective Role of Vitamin C in the Management of COVID-19: A Review. J Egypt Public Health Assoc (2021) 96:33. doi: 10.1186/s42506-021-00095-w 34894332 PMC8665316

[127] Bendich A Machlin LJ Scandurra O Burton GW Wayner DDM. The Antioxidant Role of Vitamin C. Adv Free Radic Biol Med (1986) 2:419–44. doi: 10.1016/S8755-9668(86)80021-7

[128] Hemila H. Vitamin C. And SARS Coronavirus. J Antimicrob Chemother (2003) 52:1049–50. doi: 10.1093/jac/dkh002 PMC7110025 14613951

[129] Atherton JG Kratzing CC Fisher A. The Effect of Ascorbic Acid on Infection Chick-Embryo Ciliated Tracheal Organ Cultures by Coronavirus. Arch Virol (1978) 56:195–9. doi: 10.1007/BF01317848 PMC7087159 205194

[130] Field CJ Johnson IR Schley PD. Nutrients and Their Role in Host Resistance to Infection. J Leukoc Biol (2002) 71:16–32. doi: 10.1189/jlb.71.1.16 11781377

[131] Teafatiller T Agrawal S De Robles G Rahmatpanah F Subramanian VS Agrawal A. Vitamin C Enhances Antiviral Functions of Lung Epithelial Cells. Biomolecules (2021) 11:1148. doi: 10.3390/biom11081148 34439814 PMC8394979

[132] Holick MF. Sunlight and Vitamin D for Bone Health and Prevention of Autoimmune Diseases, Cancers, and Cardiovascular Disease. Am J Clin Nutr (2004) 80:1678S–88S. doi: 10.1093/ajcn/80.6.1678S 15585788

[133] Holick MF. High Prevalence of Vitamin D Inadequacy and Implications for Health. Mayo Clin Proc (2006) 81:353–73. doi: 10.4065/81.3.353 16529140

[134] Beard JA Bearden A Striker R. Vitamin D and the Anti-Viral State. J Clin Virol (2011) 50:194–200. doi: 10.1016/j.jcv.2010.12.006 21242105 PMC3308600

[135] Luong K Nguyen LTH. Impact of Vitamin D in the Treatment of Tuberculosis. Am J Med Sci (2011) 341:493–8. doi: 10.1097/MAJ.0b013e3182070f47 21289501

[136] Martineau AR Jolliffe DA Hooper RL Greenberg L Aloia JF Bergman P. Vitamin D Supplementation to Prevent Acute Respiratory Tract Infections: Systematic Review and Meta-Analysis of Individual Participant Data. BMJ (2017) 356:i6583. doi: 10.1136/bmj.i6583 28202713 PMC5310969

[137] Bauer SR Kapoor A Rath M Thomas SA. What Is the Role of Supplementation With Ascorbic Acid, Zinc, Vitamin D, or N -Acetylcysteine for Prevention or Treatment of COVID-19? Cleve Clin J Med (2020), 1–3. doi: 10.3949/ccjm.87a.ccc046 32513807

[138] Linneberg A Kampmann FB Israelsen SB Andersen LR Jørgensen HL Sandholt H. The Association of Low Vitamin K Status With Mortality in a Cohort of 138 Hospitalized Patients With COVID-19. Nutrients (2021) 13:1985. doi: 10.3390/nu13061985 34207745 PMC8229962

[139] Janssen R Visser MPJ Dofferhoff ASM Vermeer C Janssens W Walk J. Vitamin K Metabolism as the Potential Missing Link Between Lung Damage and Thromboembolism in Coronavirus Disease 2019. Br J Nutr (2020) 126:191–8. doi: 10.1017/S0007114520003979 PMC7578635 33023681

[140] Melhus H. Excessive Dietary Intake of Vitamin A Is Associated With Reduced Bone Mineral Density and Increased Risk for Hip Fracture. Ann Intern Med (1998) 129:770. doi: 10.7326/0003-4819-129-10-199811150-00003 9841582

[141] Slatore CG Littman AJ Au DH Satia JA White E. Long-Term Use of Supplemental Multivitamins, Vitamin C, Vitamin E, and Folate Does Not Reduce the Risk of Lung Cancer. Am J Respir Crit Care Med (2008) 177:524–30. doi: 10.1164/rccm.200709-1398OC PMC2258445 17989343

[142] Ebbing M. Cancer Incidence and Mortality After Treatment With Folic Acid and Vitamin B12. JAMA (2009) 302:2119. doi: 10.1001/jama.2009.1622 19920236

[143] Kesse-Guyot E Bertrais S Duperray B Arnault N Bar-Hen A Galan P. Dairy Products, Calcium and the Risk of Breast Cancer: Results of the French SU.VI.MAX Prospective Study. Ann Nutr Metab (2007) 51:139–45. doi: 10.1159/000103274 17536191

[144] McCullough ML. Dairy, Calcium, and Vitamin D Intake and Postmenopausal Breast Cancer Risk in the Cancer Prevention Study II Nutrition Cohort. Cancer Epidemiol Biomark Prev (2005) 14:2898–904. doi: 10.1158/1055-9965.EPI-05-0611 16365007

[145] Liu J Cao R Xu M Wang X Zhang H Hu H. Hydroxychloroquine, a Less Toxic Derivative of Chloroquine, Is Effective in Inhibiting SARS-CoV-2 Infection *in vitro* . Cell Discov (2020) 6:16. doi: 10.1038/s41421-020-0156-0 32194981 PMC7078228

[146] Savarino A Di Trani L Donatelli I Cauda R Cassone A. New Insights Into the Antiviral Effects of Chloroquine. Lancet Infect Dis (2006) 6:67–9. doi: 10.1016/S1473-3099(06)70361-9 PMC7129107 16439323

[147] Dong L Hu S Gao J. Discovering Drugs to Treat Coronavirus Disease 2019 (COVID-19). Drug Discov Ther (2020) 14:58–60. doi: 10.5582/ddt.2020.01012 32147628

[148] Hendaus MA. Remdesivir in the Treatment of Coronavirus Disease 2019 (COVID-19): A Simplified Summary. J Biomol Struct Dyn (2021) 39:3787–92. doi: 10.1080/07391102.2020.1767691 PMC7256348 32396771

[149] Stebbing J Phelan A Griffin I Tucker C Oechsle O Smith D. COVID-19: Combining Antiviral and Anti-Inflammatory Treatments. Lancet Infect Dis (2020) 20:400–2. doi: 10.1016/S1473-3099(20)30132-8 PMC7158903 32113509

[150] Choy K-T Wong AY-L Kaewpreedee P Sia SF Chen D Hui KPY. Remdesivir, Lopinavir, Emetine, and Homoharringtonine Inhibit SARS-CoV-2 Replication *In Vitro* . Antiviral Res (2020) 178:104786. doi: 10.1016/j.antiviral.2020.104786 32251767 PMC7127386

[151] Venisse N Peytavin G Bouchet S Gagnieu M-C Garraffo R Guilhaumou R. Concerns About Pharmacokinetic (PK) and Pharmacokinetic-Pharmacodynamic (PK-PD) Studies in the New Therapeutic Area of COVID-19 Infection. Antiviral Res (2020) 181:104866. doi: 10.1016/j.antiviral.2020.104866 32659293 PMC7351053

[152] Acquavia MA Foti L Pascale R Nicolò A Brancaleone V Cataldi TRI. Detection and Quantification of Covid-19 Antiviral Drugs in Biological Fluids and Tissues. Talanta (2021) 224:121862. doi: 10.1016/j.talanta.2020.121862 33379073 PMC7642756

[153] Khadka S Yuchi A Shrestha DB Budhathoki P Al-Subari SMM Ziad Alhouzani TM. Repurposing Drugs for COVID-19: An Approach for Treatment in the Pandemic. Altern Ther Health Med (2020) 26:100–7. 32827400

[154] Parvathaneni V Gupta V. Utilizing Drug Repurposing Against COVID-19 – Efficacy, Limitations, and Challenges. Life Sci (2020) 259:118275. doi: 10.1016/j.lfs.2020.118275 32818545 PMC7430345

[155] Demeke CA Woldeyohanins AE Kifle ZD. Herbal Medicine Use for the Management of COVID-19: A Review Article. Metab Open (2021) 12:100141. doi: 10.1016/j.metop.2021.100141 PMC8519661 34693242

[156] Sanders JM Monogue ML Jodlowski TZ Cutrell JB. Pharmacologic Treatments for Coronavirus Disease 2019 (COVID-19). JAMA (2020) 323:1824–36. doi: 10.1001/jama.2020.6019 32282022

[157] Gbinigie K Frie K. Should Chloroquine and Hydroxychloroquine be Used to Treat COVID-19? A Rapid Review. BJGP Open (2020) 4:bjgpopen20X101069. doi: 10.3399/bjgpopen20X101069 PMC7330219 32265182

[158] Low ZY Farouk IA Lal SK. Drug Repositioning: New Approaches and Future Prospects for Life-Debilitating Diseases and the COVID-19 Pandemic Outbreak. Viruses (2020) 12:1058. doi: 10.3390/v12091058 PMC7551028 32972027

[159] Barlow A Landolf KM Barlow B Yeung SYA Heavner JJ Claassen CW. Review of Emerging Pharmacotherapy for the Treatment of Coronavirus Disease 2019. Pharmacother J Hum Pharmacol Drug Ther (2020) 40:416–37. doi: 10.1002/phar.2398 PMC7262196 32259313

[160] Yasui H Shida K Matsuzaki T Yokokura T. Immunomodulatory Function of Lactic Acid Bacteria. Antonie Van Leeuwenhoek. Int J Gen Mol Microbiol (1999) 76:383–9. doi: 10.1023/A:1002041616085 10532394

[161] Hori T Kivoshima J Shida K Yasui H. Augmentation of Cellular Immunity and Reduction of Influenza Virus Titer in Aged Mice Fed Lactobacillus Casei Strain Shirota. Clin Diagn Lab Immunol (2002) 9:105–8. doi: 10.1128/CDLI.9.1.105-108.2002 PMC119906 11777838

[162] Hatakka K Savilahti E Pönkä A Meurman JH Poussa T Näse L. Effect of Long Term Consumption of Probiotic Milk on Infections in Children Attending Day Care Centres: Double Blind, Randomised Trial. Br Med J (2001) 322:1327–9. doi: 10.1136/bmj.322.7298.1327 PMC32161 11387176

[163] Hossain KS Hossain MG Moni A Rahman MM Rahman UH Alam M. Prospects of Honey in Fighting Against COVID-19: Pharmacological Insights and Therapeutic Promises. Heliyon (2020) 6:e05798. doi: 10.1016/j.heliyon.2020.e05798 33363261 PMC7750705

[164] Poland GA Ovsyannikova IG Kennedy RB. SARS-CoV-2 Immunity: Review and Applications to Phase 3 Vaccine Candidates. Lancet (2020) 396:1595–606. doi: 10.1016/S0140-6736(20)32137-1 PMC7553736 33065034

[165] Ball DW. The Chemical Composition of Honey. J Chem Educ (2007) 84:1643–6. doi: 10.1021/ED084P1643

[166] Cheng N Wang Y Cao W. The Protective Effect of Whole Honey and Phenolic Extract on Oxidative DNA Damage in Mice Lymphocytes Using Comet Assay. Plant Foods Hum Nutr (2017) 72:388–95. doi: 10.1007/S11130-017-0634-1 28929426

[167] Dżugan M Sowa P Kwaśniewska M Wesołowska M Czernicka M. Physicochemical Parameters and Antioxidant Activity of Bee Honey Enriched With Herbs. Plant Foods Hum Nutr (2017) 72:74–81. doi: 10.1007/s11130-016-0593-y 28000091

[168] Sulaiman SA Hasan H Deris Z Wahad MS Yousof RC Naing N. The Benefit of Tualang Honey in Reducing Acute Respiratory Symptoms Among Malaysian Hajj Pilgrims: A Preliminary Study. J Apiproduct Apimed Sci (2011) 3:38–44. doi: 10.3896/IBRA.4.03.1.07

[169] Wessels I Rolles B Rink L. The Potential Impact of Zinc Supplementation on COVID-19 Pathogenesis. Front Immunol (2020) 11:1712. doi: 10.3389/fimmu.2020.01712 32754164 PMC7365891

[170] Jothimani D Kailasam E Danielraj S Nallathambi B Ramachandran H Sekar P. COVID-19: Poor Outcomes in Patients With Zinc Deficiency. Int J Infect Dis (2020) 100:343–9. doi: 10.1016/j.ijid.2020.09.014 PMC7482607 32920234

[171] Maywald M Wessels I Rink L. Zinc Signals and Immunity. Int J Mol Sci (2017) 18:2222. doi: 10.3390/ijms18102222 PMC5666901 29064429

[172] Bouvet M Debarnot C Imbert I Selisko B Snijder EJ Canard B. *In Vitro* Reconstitution of SARS-Coronavirus mRNA Cap Methylation. PloS Pathog (2010) 6:e1000863. doi: 10.1371/journal.ppat.1000863 20421945 PMC2858705

[173] Asl SH Nikfarjam S Majidi Zolbanin N Nassiri R Jafari R. Immunopharmacological Perspective on Zinc in SARS-CoV-2 Infection. Int Immunopharmacol (2021) 96:107630. doi: 10.1016/j.intimp.2021.107630 33882442 PMC8015651

[174] Vogel-González M Talló-Parra M Herrera-Fernández V Pérez-Vilaró G Chillón M Nogués X. Low Zinc Levels at Admission Associates With Poor Clinical Outcomes in SARS-CoV-2 Infection. Nutrients (2021) 13:562. doi: 10.3390/nu13020562 33572045 PMC7914437

[175] Razzaque MS. COVID-19 Pandemic: Can Zinc Supplementation Provide an Additional Shield Against the Infection? Comput Struct Biotechnol J (2021) 19:1371–8. doi: 10.1016/j.csbj.2021.02.015 PMC7923946 33680350

[176] Quilliot D Bonsack O Jaussaud R Mazur A. Dysmagnesemia in Covid-19 Cohort Patients: Prevalence and Associated Factors. Magnes Res (2020) 33:114–22. doi: 10.1684/mrh.2021.0476 33678604

[177] Tang C-F Ding H Jiao R-Q Wu X-X Kong L-D. Possibility of Magnesium Supplementation for Supportive Treatment in Patients With COVID-19. Eur J Pharmacol (2020) 886:173546. doi: 10.1016/j.ejphar.2020.173546 32931782 PMC7486870

[178] Dominguez LJ Veronese N Guerrero-Romero F Barbagallo M. Magnesium in Infectious Diseases in Older People. Nutrients (2021) 13:180. doi: 10.3390/nu13010180 PMC7827130 33435521

[179] Uwitonze AM. Razzaque MS.Role of Magnesium in Vitamin D Activation Andfunction. J Am Osteopath Assoc (2018) 118:181–9. doi: 10.7556/jaoa.2018.037 29480918

[180] Tan CW Ho LP Kalimuddin S Cherng BPZ Teh YE Thien SY. A Cohort Study to Evaluate the Effect of Combination Vitamin D, Magnesium and Vitamin B12 on Progression to Severe Outcome in Older COVID-19 Patients. Nutrition (2020) 79-80:111017. doi: 10.1016/j.nut.2020.111017 33039952 PMC7832811

[181] DiNicolantonio JJ O’Keefe JH. Magnesium and Vitamin D Deficiency as a Potential Cause of Immune Dysfunction, Cytokine Storm and Disseminated Intravascular Coagulation in Covid-19 Patients. Mo Med (2021) 118:68–73. 33551489 PMC7861592

[182] Maggini S Pierre A Calder P. Immune Function and Micronutrient Requirements Change Over the Life Course. Nutrients (2018) 10:1531. doi: 10.3390/nu10101531 PMC6212925 30336639

[183] Percival SS. Copper and Immunity. Am J Clin Nutr (1998) 67:1064S–8S. doi: 10.1093/ajcn/67.5.1064S 9587153

[184] Besold AN Culbertson EM Culotta VC. The Yin and Yang of Copper During Infection. JBIC J Biol Inorg Chem (2016) 21:137–44. doi: 10.1007/s00775-016-1335-1 PMC5535265 26790881

[185] Warnes SL Little ZR Keevil CW. Human Coronavirus 229e Remains Infectious on Common Touch Surface Materials. MBio (2015) 6:1–15. doi: 10.1128/mBio.01697-15 PMC4659470 26556276

[186] Mao S Zhang A Huang S. Meta-Analysis of Zn, Cu and Fe in the Hair of Chinese Children With Recurrent Respiratory Tract Infection. Scand J Clin Lab Invest (2014) 74:561–7. doi: 10.3109/00365513.2014.921323 24874085

[187] Huang Z Rose AH Hoffmann PR. The Role of Selenium in Inflammation and Immunity: From Molecular Mechanisms to Therapeutic Opportunities. AntioxidRedox Signal (2012) 16:705–43. doi: 10.1089/ars.2011.4145 PMC3277928 21955027

[188] Arthur JR McKenzie RC Beckett GJ. Selenium in the Immune System. J Nutr (2003) 133:1457S–9S. doi: 10.1093/jn/133.5.1457S 12730442

[189] McKenzie RC S Rafferty T Beckett GJ. Selenium: An Essential Element for Immune Function. Immunol Today (1998) 19:342–5. doi: 10.1016/S0167-5699(98)01294-8 9709500

[190] Peretz A Nève J Desmedt J Duchateau J Dramaix M Famaey JP. Lymphocyte Response Is Enhanced by Supplementation of Elderly Subjects With Selenium-Enriched Yeast. Am J Clin Nutr (1991) 53:1323–8. doi: 10.1093/ajcn/53.5.1323 2021141

[191] Roy M Kiremidjian-Schumacher L Wishe HI Cohen MW Stotzky G. Supplementation With Selenium and Human Immune Cell Functions: I. Effect on Lymphocyte Proliferation and Interleukin 2 Receptor Expression. Biol Trace Elem Res (1994) 46:183–3. doi: 10.1007/BF02790078 7946898

[192] Auinger A Riede L Bothe G Busch R Gruenwald J. Yeast (1,3)-(1,6)-Beta-Glucan Helps to Maintain the Body’s Defence Against Pathogens: A Double-Blind, Randomized, Placebo-Controlled, Multicentric Study in Healthy Subjects. Eur J Nutr (2013) 52:1913–8. doi: 10.1007/s00394-013-0492-z PMC3832763 23340963

[193] Graubaum H-J Busch R Stier H Gruenwald J. A Double-Blind, Randomized, Placebo-Controlled Nutritional Study Using an Insoluble Yeast Beta-Glucan to Improve the Immune Defense System. Food Nutr Sci (2012) 03:738–46. doi: 10.4236/fns.2012.36100

[194] McFarlin BK Carpenter KC Davidson T McFarlin MA. Baker’s Yeast Beta Glucan Supplementation Increases Salivary IgA and Decreases Cold/Flu Symptomatic Days After Intense Exercise. J Diet Suppl (2013) 10:171–83. doi: 10.3109/19390211.2013.820248 PMC5044766 23927572

[195] Mahn A Castillo A. Potential of Sulforaphane as a Natural Immune System Enhancer: A Review. Molecules (2021) 26:752. doi: 10.3390/molecules26030752 33535560 PMC7867070

[196] Thota SM Balan V Sivaramakrishnan V. Natural Products as Home-Based Prophylactic and Symptom Management Agents in the Setting of COVID -19. Phyther Res (2020) 34:3148–67. doi: 10.1002/ptr.6794 PMC7461159 32881214

[197] Levy M Thaiss CA Elinav E. Metabolites: Messengers Between the Microbiota and the Immune System. Genes Dev (2016) 30:1589–97. doi: 10.1101/gad.284091.116 PMC4973288 27474437

[198] Bartolini D Stabile AM Bastianelli S Giustarini D Pierucci S Busti C. SARS-CoV2 Infection Impairs the Metabolism and Redox Function of Cellular Glutathione. Redox Biol (2021) 45:102041. doi: 10.1016/j.redox.2021.102041 34146958 PMC8190457

[199] Thaker SK Chng J Christofk HR. Viral hijacking of cellular metabolism. BMC Biol (2019) 17:1–15. doi: 10.1186/s12915-019-0678-9 31319842 PMC6637495

[200] Venegas DP de la Fuente MK Landskron G González MJ Quera R Dijkstra G. Short Chain Fatty Acids (SCFAs)mediated Gut Epithelial and Immune Regulation and its Relevance for Inflammatory Bowel Diseases. Front Immunol (2019) 10:277. doi: 10.3389/fimmu.2019.00277 30915065 PMC6421268

[201] den Besten G van Eunen K Groen AK Venema K Reijngoud D-J Bakker BM. The Role of Short-Chain Fatty Acids in the Interplay Between Diet, Gut Microbiota, and Host Energy Metabolism. J Lipid Res (2013) 54:2325–40. doi: 10.1194/jlr.R036012 PMC3735932 23821742

[202] Hubbard TD Murray IA Perdew GH. Special Section on Drug Metabolism and the Microbiome - Minireview Indole and Tryptophan Metabolism: Endogenous and Dietary Routes to Ah Receptor Activation. Drug Metab Dispos (2015) 43:1522–35. doi: 10.1124/dmd.115.064246 PMC4576673 26041783

[203] Qiu J Guo X Chen ZE He L Sonnenberg GF Artis D. Group 3 Innate Lymphoid Cells Inhibit T-Cell-Mediated Intestinal Inflammation Through Aryl Hydrocarbon Receptor Signaling and Regulation of Microflora. Immunity (2013) 39:386–99. doi: 10.1016/j.immuni.2013.08.002 PMC3884586 23954130

[204] Li Y Innocentin S Withers DR Roberts NA Gallagher AR Grigorieva EF. Exogenous Stimuli Maintain Intraepithelial Lymphocytes *via* Aryl Hydrocarbon Receptor Activation. Cell (2011) 147:629–40. doi: 10.1016/j.cell.2011.09.025 21999944

[205] Balić A Vlašić D Žužul K Marinović B Bukvić Mokos Z. Omega-3 Versus Omega-6 Polyunsaturated Fatty Acids in the Prevention and Treatment of Inflammatory Skin Diseases. Int J Mol Sci (2020) 21:741. doi: 10.3390/ijms21030741 PMC7037798 31979308

[206] Morita M Kuba K Ichikawa A Nakayama M Katahira J Iwamoto R. The Lipid Mediator Protectin D1 Inhibits Influenza Virus Replication and Improves Severe Influenza. Cell (2013) 153:112–25. doi: 10.1016/j.cell.2013.02.027 23477864

[207] Lao X Liu M Chen J Zheng H. A Tumor-Penetrating Peptide Modification Enhances the Antitumor Activity of Thymosin Alpha 1. PloS One (2013) 8:e72242. doi: 10.1371/journal.pone.0072242 23977262 PMC3747120

[208] Li J Liu CH Wang FS. Thymosin Alpha 1: Biological Activities, Applications and Genetic Engineering Production. Peptides (2010) 31:2151–8. doi: 10.1016/j.peptides.2010.07.026 PMC7115394 20699109

[209] Matteucci C Minutolo A Balestrieri E Petrone V Fanelli M Malagnino V. Thymosin Alpha 1 Mitigates Cytokine Storm in Blood Cells From Coronavirus Disease 2019 Patients. Open Forum Infect Dis (2021) 8. doi: 10.1093/ofid/ofaa588 PMC7798699 33506065

[210] Goldstein G Audhya TK. Thymopoietin to Thymopentin: Experimental Studies. Surv Immunol Res (1985) 4:1. doi: 10.1007/BF02919050 2994196

[211] Tan Y Wang W Wu C Pan Z Yao G Fang L. Myristic Acid-Modified Thymopentin for Enhanced Plasma Stability and Immune-Modulating Activity. Int Immunopharmacol (2017) 47:88–94. doi: 10.1016/j.intimp.2017.03.025 28365509

[212] Joffe MI Sukha NR Rabson AR. Lymphocyte Subsets in Measles. Depressed Helper/Inducer Subpopulation Reversed by *In Vitro* Treatment With Levamisole and Ascorbic Acid. J Clin Invest (1983) 72:971–80. doi: 10.1172/JCI111069 PMC1129263 6604071

[213] Devaux CA Melenotte C Piercecchi-Marti M-D Delteil C Raoult D. Cyclosporin A: A Repurposable Drug in the Treatment of COVID-19? Front Med (2021) 8:663708. doi: 10.3389/fmed.2021.663708 PMC8450353 34552938

[214] de Wilde AH Zevenhoven-Dobbe JC van der Meer Y Thiel V Narayanan K Makino S. Cyclosporin A Inhibits the Replication of Diverse Coronaviruses. J Gen Virol (2011) 92:2542–8. doi: 10.1099/vir.0.034983-0 PMC3352363 21752960

[215] Bermon S Castell LM Calder PC Bishop NC Blomstrand E Mooren FC. Consensus Statement Immunonutrition and Exercise. Exercise Immunol Rev (2017) 23:8–50. 28224969

[216] Wedell-Neergaard A-S Lang Lehrskov L Christensen RH Legaard GE Dorph E Larsen MK. Exercise-Induced Changes in Visceral Adipose Tissue Mass Are Regulated by IL-6 Signaling: A Randomized Controlled Trial. Cell Metab (2019) 29:844–55.e3. doi: 10.1016/j.cmet.2018.12.007 30595477

[217] Laddu DR Lavie CJ Phillips SA Arena R. Physical Activity for Immunity Protection: Inoculating Populations With Healthy Living Medicine in Preparation for the Next Pandemic. Prog Cardiovasc Dis (2020) 64:102–4. doi: 10.1016/j.pcad.2020.04.006 PMC7195025 32278694

[218] Duggal NA Niemiro G Harridge SDR Simpson RJ Lord JM. Can Physical Activity Ameliorate Immunosenescence and Thereby Reduce Age-Related Multi-Morbidity? Nat Rev Immunol (2019) 19:563–72. doi: 10.1038/s41577-019-0177-9 31175337

[219] Martin SA Pence BD Woods JA. Exercise and Respiratory Tract Viral Infections. Exerc Sport Sci Rev (2009) 37:157–64. doi: 10.1097/JES.0b013e3181b7b57b PMC2803113 19955864

[220] WHO Guidelines on Physical Activity and Sedentary Behaviour. Geneva:World Health Organization (2020) Licence: CC BY-NC-SA 3.0 IGO.

[221] Irwin MR Opp MR. Sleep Health: Reciprocal Regulation of Sleep and Innate Immunity. Neuropsychopharmacology (2017) 42:129–55. doi: 10.1038/npp.2016.148 PMC5143488 27510422

[222] Chaput J-P Dutil C Sampasa-Kanyinga H. Sleeping Hours: What is the Ideal Number and How Does Age Impact This? Nat Sci Sleep (2018) 10:421–30. doi: 10.2147/NSS.S163071 PMC6267703 30568521

[223] Cohen S. Psychosocial Vulnerabilities to Upper Respiratory Infectious Illness: Implications for Susceptibility to Coronavirus Disease 2019 (COVID-19). Perspect Psychol Sci (2021) 16:161–74. doi: 10.1177/1745691620942516 PMC7345443 32640177

[224] Alirezaei M Kemball CC Flynn CT Wood MR Whitton JL Kiosses WB. Short-Term Fasting Induces Profound Neuronal Autophagy. Autophagy (2010) 6:702–10. doi: 10.4161/auto.6.6.12376 PMC3106288 20534972

[225] Bagherniya M Butler AE Barreto GE Sahebkar A. The Effect of Fasting or Calorie Restriction on Autophagy Induction: A Review of the Literature. Ageing Res Rev (2018) 47:183–97. doi: 10.1016/j.arr.2018.08.004 30172870

[226] Faris “Mo’ez Al-Islam” E Kacimi S Al-Kurd RA Fararjeh MA Bustanji YK Mohammad MK. Intermittent Fasting During Ramadan Attenuates Proinflammatory Cytokines and Immune Cells in Healthy Subjects. Nutr Res (2012) 32:947–55. doi: 10.1016/j.nutres.2012.06.021 23244540

[227] Romero M del M Fernández-López J Esteve M Alemany M. Different Modulation by Dietary Restriction of Adipokine Expression in White Adipose Tissue Sites in the Rat. Cardiovasc Diabetol (2009) 8:42. doi: 10.1186/1475-2840-8-42 19642981 PMC3224727

[228] Mousa HAL. Prevention and Treatment of Influenza, Influenza-Like Illness, and Common Cold by Herbal, Complementary, and Natural Therapies. J Evid Based Complement Altern Med (2017) 22:166–74. doi: 10.1177/2156587216641831 PMC5871211 27055821

[229] Gawade AE Bale SR. A Nutritional Intervention Against Covid-19: Possibilities on the Use of an Alkaline Diet to Boost Physiological Resistance and Immunity. Indian J Tradit Knowl (2020) 19:S158–63.

[230] Taneja MK. Modified Bhramari Pranayama in Covid 19 Infection. Indian J Otolaryngol Head Neck Surg (2020) 72:395–7. doi: 10.1007/s12070-020-01883-0 PMC7239502 32719737

[231] Tyagi SC Singh M. Multi-Organ Damage by Covid-19: Congestive (Cardio-Pulmonary) Heart Failure, and Blood-Heart Barrier Leakage. Mol Cell Biochem (2021) 476:1891–5. doi: 10.1007/s11010-021-04054-z PMC7822399 33483858

[232] Torales J O’Higgins M Castaldelli-Maia JM Ventriglio A. The Outbreak of COVID-19 Coronavirus and its Impact on Global Mental Health. Int J Soc Psychiatry (2020) 66:317–20. doi: 10.1177/0020764020915212 32233719

[233] Ji D Ji YJ Duan XZ Li WG Sun ZQ Song XA. Prevalence of Psychological Symptoms Among Ebola Survivors and Healthcare Workers During the 2014-2015 Ebola Outbreak in Sierra Leone: A Cross-Sectional Study. Oncotarget (2017) 8:12784–91. doi: 10.18632/oncotarget.14498 PMC5355054 28061463

[234] Mohindra R Ravki R Suri V Bhalla A Singh SM. Issues Relevant to Mental Health Promotion in Frontline Health Care Providers Managing Quarantined/Isolated COVID19 Patients. Asian J Psychiatr (2020) 51:102084. doi: 10.1016/j.ajp.2020.102084 32289728 PMC7138416

[235] Xiao H Zhang Y Kong D Li S Yang N. The Effects of Social Support on Sleep Quality of Medical Staff Treating Patients With Coronavirus Disease 2019 (COVID-19) in January and February 2020 in China. Med Sci Monit (2020) 26. doi: 10.12659/MSM.923549 PMC7075079 32132521

[236] Silver R Kriegsfeld LJ. Circadian Rhythms Have Broad Implications for Understanding Brain and Behavior. Eur J Neurosci (2014) 39:1866–80. doi: 10.1111/ejn.12593 PMC4385795 24799154

[237] Jagannath A Taylor L Wakaf Z Vasudevan SR Foster RG. The Genetics of Circadian Rhythms, Sleep and Health. Hum Mol Genet (2017) 26:R128–38. doi: 10.1093/hmg/ddx240 PMC5886477 28977444

[238] Gopal SG. A Preliminary Report on Plant Based Immunity Against SARS-CoV-2 (COVID-19) in Pandemic 2020. Res J Biotechnol (2020) 15:174–6.

[239] Du Y Chen X. Favipiravir: Pharmacokinetics and Concerns About Clinical Trials for 2019-Ncov Infection. Clin Pharmacol Ther (2020) 108:242–7. doi: 10.1002/cpt.1844 32246834

[240] Asai A Konno M Ozaki M Otsuka C Vecchione A Arai T. COVID-19 Drug Discovery Using Intensive Approaches. Int J Mol Sci (2020) 21:2839. doi: 10.3390/ijms21082839 PMC7215413 32325767

[241] Sisay M. 3clpro Inhibitors as a Potential Therapeutic Option for COVID-19: Available Evidence and Ongoing Clinical Trials. Pharmacol Res (2020) 156:104779. doi: 10.1016/j.phrs.2020.104779 32247821 PMC7128271

[242] Elfiky AA. Ribavirin, Remdesivir, Sofosbuvir, Galidesivir, and Tenofovir Against SARS-CoV-2 RNA Dependent RNA Polymerase (RdRp): A Molecular Docking Study. Life Sci (2020) 253:117592. doi: 10.1016/j.lfs.2020.117592 32222463 PMC7102646

[243] Prasad AS. Zinc in Human Health: Effect of Zinc on Immune Cells. Mol Med (2008) 14:353–7. doi: 10.2119/2008-00033 PMC2277319 18385818

[244] Wessels I Maywald M Rink L. Zinc as a Gatekeeper of Immune Function. Nutrients (2017) 9:1–44. doi: 10.3390/nu9121286 PMC5748737 29186856

[245] Zarocostas J. How to Fight an Infodemic. Lancet (2020) 395:676. doi: 10.1016/S0140-6736(20)30461-X 32113495 PMC7133615

