# Supplementary material for: A Review on Measures to Rejuvenate Immune System: Natural Mode of Protection Against Coronavirus Infection
Source: Front Immunol. 2022 Mar 15;13:837290. doi: 10.3389/fimmu.2022.837290 (PMC8965011; doi:10.3389/fimmu.2022.837290)
Supplement: Supplementary file 1 [file DataSheet_1.pdf]

A Review on Measures to Rejuvenate Immune System: Natural Mode of Protection against Coronavirus Infection

Md. Aminul Islam[1,2†,] Md Atiqul Haque[3,4†,] Md. Arifur Rahman[1], Foysal Hossen1, Mahin Reza[1], Abanti Barua[1], Abdullah Al Marzan[5], Tuhin Das[6], Sumit Kumar Baral[7], Cheng He[3*], Firoz Ahmed[1*], Prosun Bhattacharya[8*] and Md. Jakariya[9]

1 Department of Microbiology, Noakhali Science and Technology University, Noakhali, Bangladesh, 2 Department of Microbiology President Abdul Hamid Medical College, Karimganj, Bangladesh, 3 Key Lab of Animal Epidemiology and Zoonoses of Ministry of Agriculture and Rural Affairs, College of Veterinary Medicine, China Agricultural University, Beijing, China, 4 Department of Microbiology, Faculty of Veterinary and Animal Science, Hajee Mohammad Danesh Science and Technology University, Dinajpur, Bangladesh, 5 Department of Biochemistry and Molecular Biology, Shahjalal University of Science and Technology, Sylhet, Bangladesh, 6 Department of Microbiology, University of Chittagong, Chittagong, Bangladesh, 7 Department of Microbiology, Jagannath University, Dhaka, Bangladesh, 8 COVID-19 Research@KTH, Department of Sustainable Development, Environmental Science and Engineering, KTH Royal Institute of Technology, Stockholm, Sweden, 9 Department of Environmental Science and Management, North South University, Dhaka, Bangladesh

Supplementary Table 1. Lianhuaqingwen, a traditional Chinese Medicine formulation.

Supplementary Table 2: Functions of Vitamin-C and Vitamin-D.

Supplementary Table 3: Source of Various Vitamins from Animal, Vegetables and Fruits (141, 142).

Supplementary Table 4. Recommended Daily Allowance of Mg (198).

Supplementary Table 1. Lianhuaqingwen, a traditional Chinese Medicine formulation

Herbal medicine	Bioactive compound and extract	Part	Reference
Lianhuaqingwen (Forsythia honeysuckle)	*Forsythia suspensa* (Thunb.) Vahl (Weeping forsythia)	Fruit	(103, 104)
	Ephedra sinicaStapf (Chinese ephedra)	Stem	
	*Lonicera japonica*Thunb. (Japanese honeysuckle)	Flower	
	Isatisindigotica Fortune (Woad)	Root	
	*Mentha haplocalyx*Briq. (Mint)	Leaf and stem (menthol)	
	Dryopteris crassirhizomaNakai (Thickstemmed wood fern)	Rhizome and petiole residues	
	Rhodiola rosea L. (Golden root)	Whole herb	
	Gypsum Fibrosum (Gypsum)	Plaster	
	Pogostemoncablin (Blanco) Benth.(Patchouli)	Aboveground parts	
	*Rheum palmatum*L. (Chinese rhubarb)	Root and rhizome	
	Houttuynia cordata Thunb. (Fish mint)	Aboveground parts	
	*Glycyrrhiza uralensis*Fisch. *(Liquorice)*	Root	
	Armeniacasibirica(L.) Lam. (Siberian apricot)	Seed	

Supplementary Table 2: Functions of Vitamin-C and Vitamin-D

Functions of Vitamin-C	Functions of Vitamin-D
Increase collagen synthesis,fibroblast proliferation,keratinocyte differentiation, motility/chemotaxis,phagocytosis,ROS generation and lipid synthesis (30–35)	It could protect lungs from infection, maintain the normal function of renal epithelial barriers, and improve the functions of corneal epithelial barriers function (32–35)
It works as an antioxidant/electron donor (50–53),enhances microbial killing (54,55,57,58,70,72),helps apoptosis and clearance (71,73,74),decreases necrosis/NETosis(73,75)	Calcitriol improves the phagocytic activity of macrophages and promotes their movement.<br>It can regulate the expression of the antimicrobial proteins defensin and cathelicidin responsible for direct killing of pathogenic bacteria and inhibits the production of IFN (38–40)
It enhances antibody levels (78,83–85)	It enhances the monocyte differentiation into macrophages (33)
Vitamin-C increases cytokine production (75,77,86–94) and decreases histamine levels (56,61,95–101)	Vitamin-D Promotes the processing of antigen.<br>Helps in the down regulation of major histocompatibility complex (MHC-II) (30–33).

Supplementary Table 3: Source of Various Vitamins from Animal, Vegetables and Fruits (141, 142)

Name of Vitamins	Animal source	Vegetables & Fruits
Vitamin A	Lamb Liver, Beef Liver,Cod Liver Oil, Liver Sausage, Bluefin Tuna, Salmon, Goose Liver, Goat Cheese, Butter, Cheddar etc.	Winter Squash, Cooked Sweet Potato, Kale, Carrot, Raw Sweet Red Pepper, Swiss Chard, Carrots, Spinach, Mango, Pumpkin pie, Tomato juice, Papaya etc.
Vitamin B	Salmon, Beef, Milk, Eggs, Liver, Trout, Pork, Chicken and Turkey, Oysters, Clams and Mussels etc.	Legumes, Sunflower Seeds, Leafy Greens, broccoli, spinach, citrus fruits, avocados, banana etc.
Vitamin C	Raw Calf liver, Beef liver,Oysters Boiled Lamb brain, Cow’s milk, Chicken liver (fried)etc.	Plums, Chili Peppers, Guavas, Broccoli, Brussels Sprouts, Lemons, Oranges, Oranges, Papayas, Lychees.
Vitamin D	Salmon fish, mackerel, sardines, Egg yolks, Cod liver oil, Milk, etc.	Mushrooms, Orange juice, Soy yogurt, Ready-to-eat cereals etc.
Vitamin E	Abalone, Goose Meat, Atlantic Salmon, beef stew, lamb brain, Sausage oil, etc.	Wheat Germ Oil, Sunflower Seeds, Almonds, Hazelnut Oil, Sunflower Oil, Hazelnuts, Pine Nuts, Peanuts, Avocado, Red Sweet Pepper, Mango, etc.

Supplementary Table 4. Recommended Daily Allowance of Mg (198)

Age	Male (milligram)	Female (milligram)	Pregnancy (milligram)
≤6 month	30	30	-
7-12 month	75	75	-
1-3 year	80	80	
4-8 year	130	130	-
9-13 year	240	210	-
14-18 year	410	360[a]	400
19-30 year	400	310[a]	350
31-50 year	420	320[a]	360
≥ 51year	420	320	-

[a]Recommended daily allowance for females who are not pregnant and for females who are lactating.
